# Anthocyanin-Rich *Morus alba* L. Fruit Extract Phytosome Protects Neuronal Cells Against Oxidative Stress-Induced Cytotoxicity

**DOI:** 10.3390/foods15203664

**Published:** 2026-10-15

**Authors:** Jurairat Khongrum, Nut Palachai

**Affiliations:** 1 Multidisciplinary Research Institute, Chiang Mai University, Chiang Mai 50200, Thailand; jurairat.kh@cmu.ac.th; 2 Functional Food Research Center for Well-Being, Multidisciplinary Research Institute, Chiang Mai University, Chiang Mai 50200, Thailand; 3 Faculty of Medicine, Mahasarakham University, Maha Sarakham 44000, Thailand

**Keywords:** anthocyanins, *Morus alba* L., mulberry, phytosome, neuroprotection, oxidative stress, epigenetics, histone deacetylase, sirtuins, apoptosis, SH-SY5Y cells

## Abstract

This study investigated the neuroprotective potential of an anthocyanin-rich *Morus alba* L. fruit extract phytosome (MAP) against hydrogen peroxide (H_2_O_2_)-induced oxidative stress and cellular injury in SH-SY5Y cells. Cells were treated with the MAP before and during H_2_O_2_ exposure. Cell viability and cytotoxicity were assessed using MTT and lactate dehydrogenase (LDH) assays, respectively. Intracellular reactive oxygen species (ROS) production and malondialdehyde (MDA) accumulation were measured to evaluate oxidative stress, while antioxidant status was determined by assessing the activities of superoxide dismutase (SOD), catalase (CAT), and glutathione peroxidase (GSH-Px). Inflammatory responses were evaluated through the expression of tumor necrosis factor-α (TNF-α) and nuclear factor kappa B (NF-κB). Apoptosis-related proteins, including B-cell lymphoma 2 (Bcl-2) and Bcl-2-associated X protein (Bax), were also analyzed. Histone deacetylase (HDAC)/sirtuin 1 (SIRT1)-related responses were evaluated by determining HDAC activity and SIRT1 expression. The MAP treatment significantly improved cell viability and reduced LDH release following H_2_O_2_ exposure. These effects were accompanied by a significant reduction in HDAC activity and an increase in SIRT1 expression, which coincided with decreased ROS generation and lipid peroxidation, enhanced endogenous antioxidant defenses, attenuated expression of inflammatory mediators, and modulation of apoptosis-related signaling pathways. Collectively, these findings suggest that modulation of HDAC activity and SIRT1 expression may contribute to the protective activity of MAP, accompanied by the attenuation of oxidative stress, inflammation, and apoptosis-related responses. Further studies in animal models and humans are warranted to validate the neuroprotective potential of MAP and assess its potential as a food-derived bioactive ingredient.

## 1. Introduction

Oxidative stress is a critical pathological mechanism underlying neuronal dysfunction and the development of neurodegenerative disorders [1]. Excessive production of reactive oxygen species (ROS) disrupts cellular redox homeostasis, damages cellular macromolecules, and impairs mitochondrial function, ultimately leading to neuronal injury. In addition to direct oxidative damage, ROS can activate inflammatory signaling pathways and apoptotic cascades, thereby exacerbating neuronal death [2]. Consequently, strategies aimed at reducing oxidative stress and its downstream pathological consequences have attracted considerable attention for the prevention and management of neurodegenerative disorders.

Emerging evidence indicates that oxidative stress not only causes cellular damage but also influences epigenetic regulation [3]. Among the epigenetic mechanisms implicated in neuronal injury, histone acetylation and deacetylation have received increasing attention because of their roles in regulating the expression of genes involved in cellular survival, antioxidant defense, and inflammatory responses [4]. Histone deacetylases (HDACs) are key enzymes that remove acetyl groups from histone and non-histone proteins, leading to chromatin condensation and transcriptional repression. Increased HDAC activity has been associated with oxidative stress-induced neuronal damage, impaired cellular defense mechanisms, and enhanced susceptibility to apoptosis [5]. In contrast, sirtuin 1 (SIRT1), a nicotinamide adenine dinucleotide (NAD^+^)-dependent deacetylase, exerts protective effects by promoting antioxidant responses, suppressing inflammation, preserving mitochondrial function, and enhancing cell survival [6]. The opposing roles reported for HDAC activity and SIRT1 in cellular stress responses provide a rationale for examining both parameters when investigating potential epigenetic-related responses to antioxidant interventions. Dysregulation of the HDAC/SIRT1 axis has therefore emerged as a relevant feature of neuronal responses to oxidative stress, making HDAC activity and SIRT1 expression useful parameters for evaluating whether food-derived bioactives may modulate stress-responsive deacetylation pathways.

Natural products rich in polyphenols have been widely investigated for their ability to protect cells against oxidative damage. Among these, mulberry (*Morus alba* L.) fruit is a valuable source of anthocyanins, particularly cyanidin-3-O-glucoside (C3G), cyanidin-3-O-rutinoside (C3R), and malvidin-3-O-glucoside (M3G), which contribute to its characteristic color and biological properties [7]. *Morus alba* L. fruit was selected in the present study because of its high anthocyanin content and previously reported antioxidant, anti-inflammatory, and cytoprotective activities in both in vitro and in vivo models [8,9]. Anthocyanins can directly scavenge free radicals and may also influence intracellular signaling pathways involved in cellular defense. Recent studies have further suggested that anthocyanins may regulate epigenetic processes, including HDAC activity and SIRT1-dependent signaling, providing a rationale for investigating whether the protective effect of anthocyanin-rich *Morus alba* L. fruit extract is associated with changes in HDAC/SIRT1-related responses [10,11].

Despite these beneficial properties, the application of anthocyanins is limited by their relatively low stability and bioavailability. Anthocyanins are susceptible to degradation during processing, storage, and digestion, which can reduce their biological effectiveness [12]. Phytosome technology has been developed to improve the delivery of plant-derived bioactive compounds by forming complexes with phospholipids [13]. This approach has been shown to enhance compound stability, membrane permeability, and cellular uptake, thereby increasing biological activity. Consequently, phytosome has gained attention as a strategy for improving the efficacy of anthocyanin-rich extracts.

Although *Morus alba* L. fruit anthocyanins have been investigated for their antioxidant and anti-inflammatory activities, evidence regarding the effects of an anthocyanin-rich *Morus alba* L. fruit extract formulated as a phytosome on oxidative stress-induced neuronal injury remains limited. In particular, it remains unclear whether phytosome formulation is associated with enhanced modulation of antioxidant, inflammatory, and apoptotic responses, as well as HDAC activity and SIRT1 expression, in neuronal cells. Therefore, this study investigated the potential neuroprotective activity of *Morus alba* L. fruit extract phytosome (MAP) against hydrogen peroxide (H_2_O_2_)-induced oxidative stress and cellular injury in SH-SY5Y cells. We hypothesized that the MAP formulation would attenuate H_2_O_2_-induced cellular injury and modulate HDAC activity and SIRT1 expression, accompanied by improvements in endogenous antioxidant defenses and attenuation of inflammatory and apoptosis-related responses.

## 2. Materials and Methods

### 2.1. Preparation of MAP

*Morus alba* L. fruits (var. Chiang Mai) were obtained from the Queen Sirikit Department of Sericulture Center, Udon Thani, Thailand. Plant authentication was conducted by the center, and a voucher specimen (No. 61001) was deposited for future reference. The fruits were collected from *Morus alba* L. trees approximately 2–3 years of age during the harvest season in February 2026. Fruits at full maturity (M7) were selected based on fruit color, characterized by a uniform dark purple to black coloration, according to previously reported maturity criteria for *Morus alba* L. fruits [14]. A total of 20 trees were sampled, and approximately 50 kg of fresh *Morus alba* L. fruits were collected, of which 10 kg was used for extraction.

The fruits were thoroughly washed, dried in a hot-air oven (Memmert GmbH, Schwabach, Germany) at 45 °C for 48 h, and subsequently ground into a fine powder. The powdered material was extracted with 50% hydroalcoholic solvent (ethanol/water, *v*/*v*) using the maceration method at a solid-to-solvent ratio of 1:10 (*w*/*v*) at 25 °C for 48 h. Following extraction, the mixture was centrifuged at 3000 rpm for 10 min, and the supernatant was filtered through Whatman No. 1 filter paper. The filtrate was concentrated under reduced pressure using an IKA Rotary Evaporator (RV 3 V, IKA-Werke GmbH & Co. KG, Staufen, Germany) and subsequently freeze-dried (Labconco FreeZone Freeze Dryer, Labconco Corporation, Kansas City, MO, USA) to obtain the dried extract.

The MAP was prepared according to previously reported method. Briefly, the dried *Morus alba* L. fruit extract (MAE) was dissolved in 50% ethanol (*v*/*v*) at a concentration of 100 mg/mL, while phosphatidylcholine (PC) was separately dissolved in dichloromethane at 100 mg/mL. The MAE and PC solutions were combined at ratios of 25:75, 33:67, 50:50, 67:33, and 75:25 (*w*/*w*). The resulting mixtures were sonicated at 25–30 kHz for 2 min, with the procedure repeated three times. The mixtures were then stirred continuously using a magnetic stirrer (H3770-HS Digital Hotplate Stirrer, Benchmark Scientific, Edison, NJ, USA) at 25 °C for 8 h to promote complex formation. The suspensions were subsequently subjected to high-speed centrifugation at 10,000× *g* for 45 min using a High-Speed Centrifuge LHS-C10 (Labtron Equipment Ltd., Camberley, UK), and the organic solvents were removed using a rotary evaporator at 45 °C for 3 h. The resulting dispersion was spray-dried using a BUCHI Mini Spray Dryer (B-290, BÜCHI Labortechnik AG, Flawil, Switzerland). The dried MAP powder was stored in a desiccator containing silica gel at 4 °C until further use [15,16].

### 2.2. Physicochemical Characterization of MAP

#### 2.2.1. Determination of Total Phenolic Content (TPC) and Total Anthocyanin Content (TAC)

TPC was determined using the Folin–Ciocalteu method as described in our previous study with slight modifications. Briefly, samples were dissolved in 95% methanol prior to the assay. An aliquot of 20 μL of sample was mixed with 20 μL of 50% Folin–Ciocalteu reagent and 158 μL of distilled water and allowed to react for 8 min. Subsequently, 30 μL of 20% sodium carbonate solution was added, and the mixture was incubated in the dark at 25 °C for 2 h. Absorbance was measured at 765 nm using a Synergy H1 Hybrid Multi-Mode Reader (BioTek Instruments, Winooski, VT, USA). A corresponding sample blank containing the same amount of sample and 95% methanol but without Folin–Ciocalteu reagent was included to account for background absorbance from the sample matrix. TPC was calculated from a gallic acid standard curve and expressed as μg gallic acid equivalents (GAE)/mg extract for MAE and μg GAE/mg formulation for MAP [9].

TAC was quantified using the pH differential method as previously reported in our earlier work. Briefly, aliquots of the sample were separately diluted in potassium chloride buffer (0.025 M, pH 1.0) and sodium acetate buffer (0.4 M, pH 4.5) and allowed to equilibrate for 10 min at 25 °C. Absorbance was then recorded at 520 and 720 nm using a UV–visible spectrophotometer (Pharmacia LKB-Biochrom 4060, Uppsala, Sweden). TAC was calculated using the molecular weight (449.2 g/mol) and molar extinction coefficient (26,900 L mol^−1^ cm^−1^) of C3G and expressed as μg C3G equivalents/mg extract for MAE and μg C3G equivalents/mg formulation for MAP [9].

#### 2.2.2. Determination of Particle Size (PS), Zeta Potential (ZP), Polydispersity Index (PDI), and Anthocyanin Association Efficiency (AE)

PS, ZP, and PDI of the MAP were determined using a Zetasizer Nano ZS (Malvern Instruments Ltd., Worcestershire, UK). Samples were diluted ten-fold with distilled water and analyzed at 25 °C by dynamic light scattering (DLS). For ZP measurement, samples were sonicated for 15 min before analysis.

AE was calculated based on the amount of free anthocyanins remaining in the supernatant after centrifugation. Briefly, the MAP powder was dispersed in distilled water and thoroughly mixed to obtain a homogeneous suspension. The suspension was centrifuged at 15,000× *g* for 30 min at 4 °C to separate the phytosome-associated fraction from free anthocyanins. Following centrifugation, the supernatant was carefully collected and analyzed for TAC using the pH differential method. The AE was calculated according to the following equation:

AE (%) = [(TAC_initial_ − TAC_free_)/TAC_initial_] × 100

where TAC_initial_ represents the initial anthocyanin content before phytosome formation and TAC_free_ represents the anthocyanin content remaining in the supernatant after centrifugation. Under this operational definition, AE represents the proportion of anthocyanins not recovered in the supernatant and therefore operationally associated with the phytosome fraction.

### 2.3. Chromatographic Characterization of the Anthocyanins

The samples were analyzed by high-performance liquid chromatography with diode-array detection (HPLC-DAD). For sample preparation, 10 mg of sample was dissolved in 1 mL of methanol to obtain a stock solution at a concentration of 10 mg/mL. The resulting solution was thoroughly mixed and filtered through a 0.45 µm nylon membrane filter prior to HPLC-DAD analysis.

Chromatographic separation was performed using a Hypersil ODS column (4.0 × 250 mm, 5 µm; Agilent Technologies, Santa Clara, CA, USA) maintained at 25 °C, with a gradient elution system consisting of 0.1% formic acid in water as mobile phase A and acetonitrile as mobile phase B. The flow rate was set at 0.3 mL/min, and the injection volume was 10 µL. Anthocyanins were monitored at 520 nm. The gradient program started at 0% B and was increased to 10% B at 8 min, followed by a linear increase to 94% B at 19.5 min. The mobile phase was then maintained at 94% B until 34.5 min.

The chromatographic profile was used to qualitatively identify C3G and M3G. The HPLC conditions were adapted from a previously reported method for anthocyanin analysis, with modifications to suit the extracts analyzed in this study [17].

### 2.4. Cell Culture and Cell Viability Assay

#### 2.4.1. Cell Culture

Human neuroblastoma SH-SY5Y cells (ATCC^®^ CRL-2266™, Manassas, VA, USA) were used as an in vitro neuronal model. Cells were maintained in Dulbecco’s Modified Eagle Medium (DMEM; Gibco, Grand Island, NY, USA) supplemented with 10% fetal bovine serum (FBS), 1% penicillin–streptomycin, and 1% non-essential amino acids. Cultures were maintained at 37 °C in a humidified incubator containing 5% CO_2_. The medium was replaced every 2–3 days, and cells were passaged upon reaching approximately 80–90% confluence [18].

#### 2.4.2. Cell Viability Assay

Cell viability was assessed using the 3-(4,5-dimethylthiazol-2-yl)-2,5-diphenyltetrazolium bromide (MTT) assay. SH-SY5Y cells were seeded in 96-well plates at a density of 1 × 10^4^ cells/well and allowed to adhere overnight. To establish the oxidative stress model, cells were exposed to various concentrations of H_2_O_2_ (0–800 μM) for 24 h. Based on our preliminary experiments and previous observations, 200 μM H_2_O_2_ was selected for subsequent studies because it consistently reduced cell viability while preserving an adequate number of viable cells for evaluating protective effects [9].

To determine the cytotoxicity of MAE, PC, and MAP, SH-SY5Y cells were treated with increasing concentrations (0–3000 μg/mL) for 24 h. For neuroprotection studies, cells were pretreated with the test substances for 24 h before H_2_O_2_ challenge. Following pretreatment, the medium was replaced with fresh medium containing H_2_O_2_ together with the respective treatments, and incubation was continued for an additional 24 h.

At the end of the treatment period, MTT solution (Sigma-Aldrich, St. Louis, MO, USA) was added to each well to achieve a final concentration of 0.5 mg/mL, followed by incubation at 37 °C for 1 h. The medium was then removed, and the resulting formazan crystals were dissolved in 100 μL dimethyl sulfoxide. Absorbance was recorded at 570 nm using a Synergy H1 Hybrid Multi-Mode Reader. Cell viability was expressed as a percentage of the untreated control group [19].

### 2.5. Lactate Dehydrogenase (LDH) Release Assay

Cellular membrane damage was assessed by measuring extracellular LDH activity. SH-SY5Y cells were seeded in 96-well plates at a density of 1 × 10^4^ cells/well and treated as described above. Following treatment, aliquots of culture supernatant were collected and transferred to a new 96-well plate for analysis.

An equal volume of LDH reaction reagent was added to each sample and incubated at 37 °C for 30 min in the dark. The absorbance of the reaction product was measured at 492 nm using a Synergy H1 Hybrid Multi-Mode Reader. LDH release was normalized to the untreated control group and expressed as an indicator of membrane damage and cytotoxicity [20].

### 2.6. Measurement of Intracellular ROS Levels

Intracellular ROS production was determined using the fluorescent probe 5-(and-6)-chloromethyl-2′,7′-dichlorodihydrofluorescein diacetate acetyl ester (CM-H_2_DCFDA). SH-SY5Y cells were seeded in 96-well plates and cultured under standard conditions. After overnight attachment, the cells were pretreated with the indicated treatments for 24 h and then incubated with 10 μM CM-H_2_DCFDA at 37 °C for 30 min in the dark. Excess probe was removed by washing the cells with phosphate-buffered saline (PBS).

After probe loading, oxidative stress was induced by exposing the cells to H_2_O_2_ in serum-free medium, with or without the indicated treatments, for an additional 24 h. Fluorescence generated by the intracellular oxidation of CM-H_2_DCFDA was measured at excitation and emission wavelengths of 488 and 520 nm, respectively. Fluorescence intensity was expressed relative to that of the untreated control group and used as an indicator of intracellular ROS levels [21].

### 2.7. Measurement of Lipid Peroxidation

Lipid peroxidation was assessed by determining malondialdehyde (MDA) levels using the thiobarbituric acid reactive substances (TBARS) assay. Cell samples were mixed with sodium dodecyl sulfate, thiobarbituric acid, and acetic acid, followed by heating at 95 °C for 10 min to promote formation of MDA-TBA adducts. The reaction mixtures were then cooled to room temperature.

The resulting chromogenic products were extracted with an n-butanol:pyridine mixture (15:1, *v*/*v*) and separated by centrifugation at 4000× *g* for 10 min. The organic phase was collected, and absorbance was measured at 532 nm using a Synergy H1 Hybrid Multi-Mode Reader. MDA concentrations were calculated from a calibration curve prepared using 1,1,3,3-tetramethoxypropane (TMP) standards (0–15 μM) [22].

### 2.8. Measurement of Antioxidant Enzyme Activities

Superoxide dismutase (SOD) activity was measured using a xanthine/xanthine oxidase system. The reaction mixture consisted of xanthine, potassium dihydrogen phosphate buffer, ethylenediaminetetraacetic acid, and cytochrome c. Following initiation of the reaction by xanthine oxidase, absorbance was recorded at 415 nm. SOD activity was calculated using standard solutions ranging from 0 to 25 U/mL [22].

Catalase (CAT) activity was determined based on the decomposition of H_2_O_2_. Cell homogenates were incubated with the reaction mixture containing H_2_O_2_, and the reaction was terminated with sulfuric acid. Potassium permanganate was subsequently added, and absorbance was measured at 490 nm. CAT activity was quantified using enzyme standards ranging from 10 to 100 U/mL [22].

Glutathione peroxidase (GSH-Px) activity was evaluated using a colorimetric assay. Cell homogenates were incubated with dithiothreitol, monosodium phosphate, sodium azide, reduced glutathione, and H_2_O_2_. After incubation, 5,5′-dithiobis-(2-nitrobenzoic acid) was added, and absorbance was measured at 412 nm. GSH-Px activity was quantified using enzyme standards ranging from 1 to 5 U/mL [22].

### 2.9. Measurement of HDAC Activity

HDAC activity was determined using a fluorometric HDAC Activity Assay Kit (ab156064; Abcam, Cambridge, MA, USA) according to the manufacturer’s protocol. Briefly, cell lysates obtained from the experimental groups were incubated with the HDAC substrate in assay buffer under the recommended reaction conditions. Trichostatin A (10 μM), a potent HDAC inhibitor, was included as an inhibitor control.

Following enzymatic incubation, the reaction was terminated using the supplied stop solution, and the developer reagent was subsequently added to generate a fluorescent signal. Fluorescence intensity was measured at excitation and emission wavelengths of 355 and 460 nm, respectively. HDAC activity was calculated according to the manufacturer’s instructions after subtraction of the corresponding background control and expressed relative to the untreated control group [23].

### 2.10. Western Blot Analysis

Following treatment, SH-SY5Y cells were harvested and lysed using Neuronal Protein Extraction Reagent (N-PER; Thermo Fisher Scientific, Waltham, MA, USA) supplemented with a protease inhibitor cocktail. The lysates were centrifuged at 10,000× *g* for 10 min at 4 °C, and the resulting supernatants were collected for protein determination by measuring absorbance at 280 nm (A280) using a NanoDrop 2000c spectrophotometer (Thermo Fisher Scientific).

Equal amounts of protein (60 μg) were mixed with loading buffer, heated at 95 °C for 5 min, and separated by sodium dodecyl sulfate–polyacrylamide gel electrophoresis (SDS-PAGE). The resolved proteins were subsequently transferred onto polyvinylidene difluoride (PVDF) membranes. Membranes were blocked with 5% skim milk prepared in Tris-buffered saline containing 0.1% Tween-20 (TBS-T) and incubated overnight at 4 °C with primary antibodies against SIRT1, nuclear factor kappa B (NF-κB), tumor necrosis factor-α (TNF-α), B-cell lymphoma 2 (Bcl-2), Bcl-2-associated X protein (Bax), and β-actin (Cell Signaling Technology, Danvers, MA, USA).

After washing with TBS-T, membranes were incubated with horseradish peroxidase (HRP)-conjugated secondary antibody for 1 h at 25 °C. Immunoreactive bands were visualized using Clarity™ Western ECL Substrate (Bio-Rad Laboratories, Hercules, CA, USA) and captured with a ChemiDoc™ MP Imaging System (Bio-Rad Laboratories). Band intensities were quantified using Image Lab software (version 6.0.0, Bio-Rad Laboratories) and normalized to β-actin. Protein expression levels were expressed relative to those of the untreated control group [24].

### 2.11. Molecular Docking

#### 2.11.1. Protein Structure and Ligand Preparation

The crystal structures of seven therapeutic target proteins, namely HDAC2 (PDB ID: 4LXZ), HDAC6 (PDB ID: 5EDU), SIRT1 (PDB ID: 4ZZJ), TNF-α (PDB ID: 2AZ5), NF-κB (PDB ID: 1VKX), Bcl-2 (PDB ID: 6O0K), and Bax (PDB ID: 1F16), were obtained from the Protein Data Bank (RCSB PDB, https://www.rcsb.org/) [25]. These crystal structures were selected because they contain well-defined ligand-binding sites and have been widely used in structure-based drug discovery studies. The chemical structures of C3G and M3G were retrieved from the PubChem database (https://pubchem.ncbi.nlm.nih.gov/) [26]. Reference ligands were selected according to the reported inhibitors of each target protein, including vorinostat (CID: 5311) and trichostatin A (CID: 444732) for HDAC2 and HDAC6, EX-527 (CID: 5113032) for SIRT1, thalidomide (CID: 5426) for TNF-α, sulindac (CID: 1548887) for NF-κB, venetoclax (CID: 49846579) for Bcl-2, and eltrombopag (CID: 135449332) for Bax. All ligand structures were converted to three-dimensional conformations and prepared using AutoDockTools 1.5.7 by adding polar hydrogen atoms, assigning Gasteiger charges, merging non-polar hydrogen atoms, and saving them in PDBQT format [27].

#### 2.11.2. Docking Protocol Validation

The reliability of the docking protocol was evaluated by re-docking the co-crystallized ligand into the corresponding binding pocket of each target protein using the same docking parameters as those employed for the investigated compounds. The predicted binding pose was superimposed on the experimentally determined crystallographic conformation, and the root-mean-square deviation (RMSD) was calculated to assess docking accuracy. An RMSD value below 2.0 Å was considered indicative of an acceptable and reliable docking protocol.

#### 2.11.3. Protein–Ligand Docking

Molecular docking was performed using AutoDock Vina version 1.2.0 to predict the binding affinity and binding modes of the investigated compounds against seven target proteins. AutoDock Vina was selected because of its high computational efficiency and reliable performance in predicting ligand–protein interactions. Docking simulations were carried out using an exhaustiveness value of 8, an energy range of 3 kcal/mol, and 100 binding modes (num_modes = 100) to ensure sufficient conformational sampling. The binding conformation with the lowest binding free energy was selected for subsequent interaction analysis.

For each target protein, the docking search space was defined around the experimentally identified ligand-binding pocket based on the position of the co-crystallized ligand. The same docking parameters were consistently applied to all target proteins throughout the study.

Protein–ligand interactions were analyzed using Discovery Studio Visualizer 2021. Two-dimensional (2D) interaction diagrams were generated to characterize molecular interactions within the binding pocket, including conventional hydrogen bonds, carbon–hydrogen bonds, hydrophobic interactions, π-related interactions, electrostatic interactions, metal coordination, and unfavorable contacts. The binding interactions of the investigated compounds were subsequently compared with those of the corresponding reference ligands to identify key amino acid residues involved in ligand recognition and binding stabilization.

### 2.12. Statistical Analysis

Data are presented as the mean ± standard error of the mean (SEM). Statistical analyses were performed using one-way analysis of variance (ANOVA) followed by Tukey’s post hoc test for multiple comparisons. Comparisons between two groups were conducted using Student’s *t*-test. A *p*-value < 0.05 was considered statistically significant. All statistical analyses were performed using IBM SPSS Statistics for Mac, version 28.0 (IBM Corp., Armonk, NY, USA).

## 3. Results

### 3.1. Physicochemical Characterization of MAP

The physicochemical properties of MAP prepared at different MAE:PC ratios are summarized in Table 1. The MAE had a TPC and TAC of 135.67 ± 3.50 µg GAE/mg extract and 72.33 ± 2.83 µg C3G equivalent/mg extract, respectively.

The incorporation of MAE into phytosome formulations resulted in changes in TPC and TAC according to the MAE:PC ratio. TPC increased from 33.67 ± 0.84 µg GAE/mg formulation in the 25:75 formulation to 98.23 ± 1.07 µg GAE/mg formulation in the 75:25 formulation. Similarly, TAC increased from 16.83 ± 0.35 to 49.33 ± 0.67 µg C3G equivalents/mg formulation across the same formulations.

AE varied among the formulations. The 25:75 and 33:67 formulations showed AEs of 85.07 ± 5.51% and 85.15 ± 3.91%, respectively, whereas the 50:50 formulation showed an AE of 82.96 ± 4.53%. Increasing the proportion of MAE further resulted in lower AEs, with values of 69.81 ± 3.23% and 54.98 ± 2.72% for the 67:33 and 75:25 formulations, respectively.

PS increased with increasing proportions of MAE. The smallest PS was observed in the 25:75 formulation (114.02 ± 1.58 nm), whereas the largest was observed in the 75:25 formulation (204.70 ± 7.35 nm). The 50:50 formulation showed a mean PS of 153.80 ± 3.51 nm.

ZP values ranged from −38.54 ± 0.32 to −18.81 ± 0.45 mV. The 50:50 formulation showed the highest absolute ZP value (−38.54 ± 0.32 mV). Beyond the 50:50 ratio, ZP values became progressively less negative as the proportion of PC decreased. PDI values ranged from 0.12 ± 0.0011 to 0.26 ± 0.0192 among the formulations.

Among the formulations evaluated, the 50:50 MAE-to-PC formulation exhibited a favorable overall physicochemical profile, with an AE of 82.96 ± 4.53%, a mean PS of 153.80 ± 3.51 nm, a PDI within the observed range, and the highest absolute ZP value (−38.54 ± 0.32 mV). Although the 25:75 and 33:67 formulations showed slightly higher AEs, the 50:50 formulation provided a favorable overall combination of anthocyanin association and physicochemical characteristics. Therefore, the 50:50 MAE: PC formulation was selected for subsequent biological investigations.

### 3.2. HPLC Analysis of Anthocyanins

For the simultaneous determination of C3G and M3G in MAE and MAP, the developed HPLC-DAD method was validated. The method showed good linearity over the concentration ranges of 0.05–10 μg/mL for C3G and 0.10–5 μg/mL for M3G, with coefficients of determination (R^2^) of 0.999 and 0.995, respectively. The corresponding regression equations for C3G and M3G were y = 7.899x − 6.848 and y = 5.442x − 1.895, respectively (Table 2). The limits of quantification (LOQs) for C3G and M3G were 0.25 and 0.50 μg/mL, respectively, whereas the limits of detection (LODs) were 0.05 and 0.10 μg/mL, respectively, demonstrating adequate sensitivity of the developed method.

Additionally, the method demonstrated good precision, with repeatability ranging from 0.08–2.10% relative standard deviation (RSD) for C3G and 1.42–2.93% RSD for M3G. Intermediate precision ranged from 0.13–2.30% RSD for C3G and 0.16–2.28% RSD for M3G. Recovery at low, medium, and high concentration levels ranged from 97.70–108.03% for C3G and 91.61–109.53% for M3G, supporting the accuracy of the analytical method under the validated conditions.

**Figure 1:**
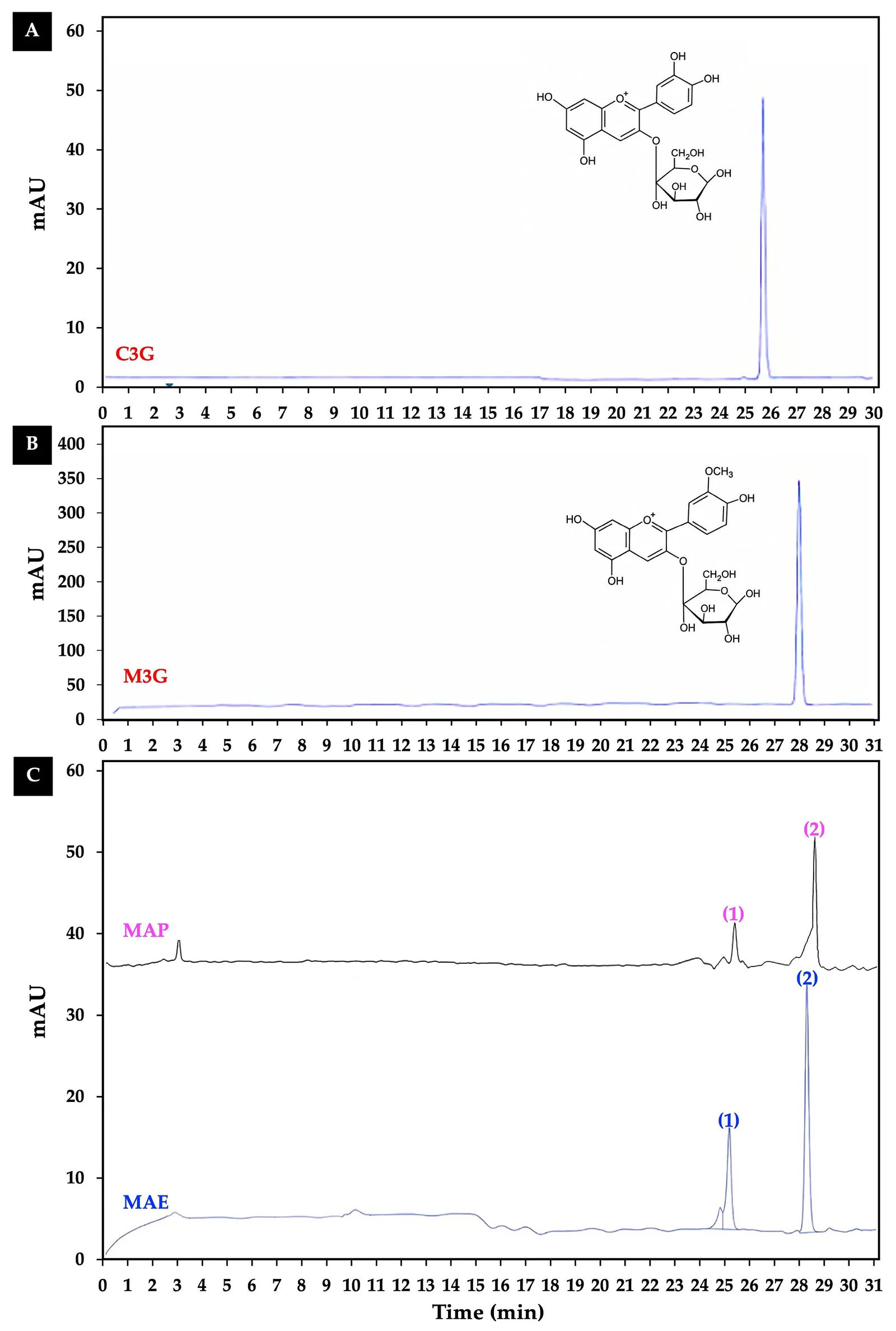
Chromatographic profiles of *Morus alba* L. fruit extract (MAE) and *Morus alba* L. fruit extract phytosome (MAP). (**A**) cyanidin-3-O-glucoside (C3G) standard; (**B**) Malvidin-3-O-glucoside (M3G) standard; and (**C**) MAE and MAP samples. Peaks correspond to (1) C3G and (2) M3G.

Under the applied sample-preparation and HPLC-DAD conditions, C3G and M3G were detected in MAE at 13.15 ± 0.04 and 38.57 ± 0.05 µg/mg extract, respectively, as shown in the chromatogram in Figure 1. In the 50:50 MAP formulation, C3G and M3G were detected at 6.72 ± 0.01 and 17.84 ± 0.02 µg/mg formulation, respectively. These values represent the amounts detected following the stated sample-preparation procedure and should therefore be interpreted as measured contents rather than as demonstrating quantitative extraction recovery from the MAP matrix.

Overall, the developed HPLC-DAD method showed adequate linearity, sensitivity, accuracy, and precision under the validated conditions for C3G and M3G analysis in MAE and MAP. Matrix-specific extraction recovery from the MAP formulation was not directly assessed in the present study.

### 3.3. Determination of Nontoxic Concentrations of MAE, PC, and MAP

**Figure 2:**
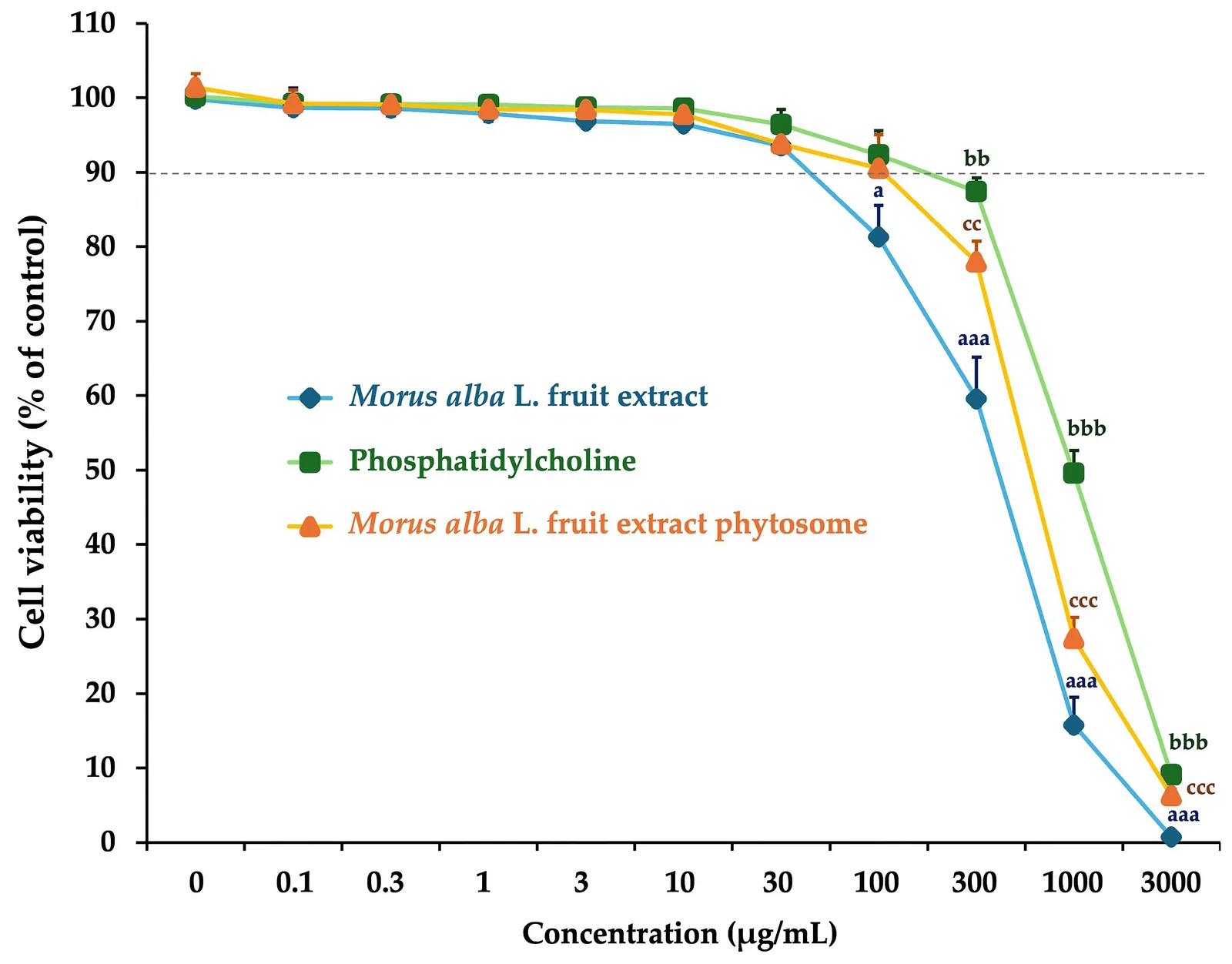
Cytotoxic effects of *Morus alba* L. fruit extract (MAE), phosphatidylcholine (PC), and *Morus alba* L. fruit extract phytosome (MAP) on SH-SY5Y cells. Cell viability was assessed using the MTT assay. Data are presented as mean ± SEM from three independent experiments (*n* = 3). Statistical analysis was performed using one-way ANOVA followed by Tukey’s post hoc test. a and aaa indicate *p* < 0.05 and *p* < 0.001, respectively, compared with the corresponding 0 μg/mL MAE group; bb and bbb indicate *p* < 0.01 and *p* < 0.001, respectively, compared with the corresponding 0 μg/mL PC group; cc and ccc indicate *p* < 0.01 and *p* < 0.001, respectively, compared with the corresponding 0 μg/mL MAP group.

The effects of MAE, PC, and MAP on SH-SY5Y cell viability were evaluated over a concentration range of 0.1–3000 μg/mL (Figure 2). Exposure to all tested preparations at concentrations up to 30 μg/mL did not significantly affect cell viability, with values remaining comparable to those of the untreated control group. A concentration-dependent reduction in cell viability was observed at higher concentrations. Treatment with MAE significantly decreased cell viability at 100 μg/mL (*p* < 0.05), with a more pronounced decline observed at 300, 1000, and 3000 μg/mL (all *p* < 0.001).

PC exhibited lower cytotoxicity than MAE, with cell viability of 92.31 ± 1.15% at 100 μg/mL. Significant reductions in cell viability were observed at 300, 1000, and 3000 μg/mL compared with the untreated control group (*p* < 0.01, *p* < 0.001, and *p* < 0.001, respectively).

Similarly, MAP maintained high cell viability at concentrations up to 100 μg/mL and showed a gradual decline at higher concentrations. Significant reductions were detected at 300, 1000, and 3000 μg/mL (*p* < 0.01, *p* < 0.001, and *p* < 0.001, respectively).

Based on these findings, concentrations that maintained more than 90% cell viability without causing a significant reduction compared with the control group were considered nontoxic concentrations. Therefore, MAE at 3, 10, and 30 μg/mL, as well as PC and MAP at 10, 30, and 100 μg/mL, were selected for subsequent experiments evaluating their protective effects against H_2_O_2_-induced oxidative stress in SH-SY5Y cells.

### 3.4. Effects of MAE, PC, and MAP on Cell Viability and LDH Release

**Figure 3:**
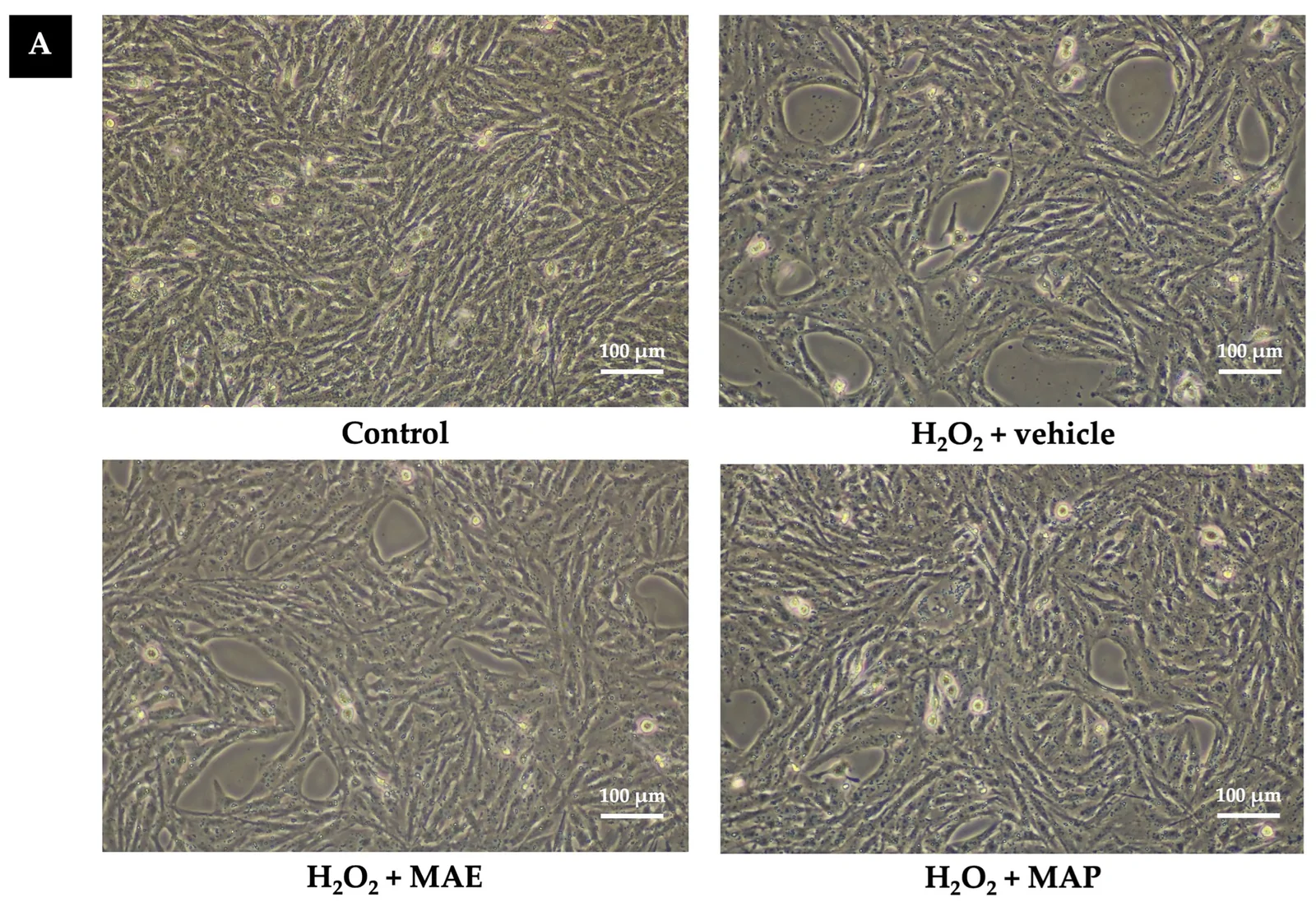

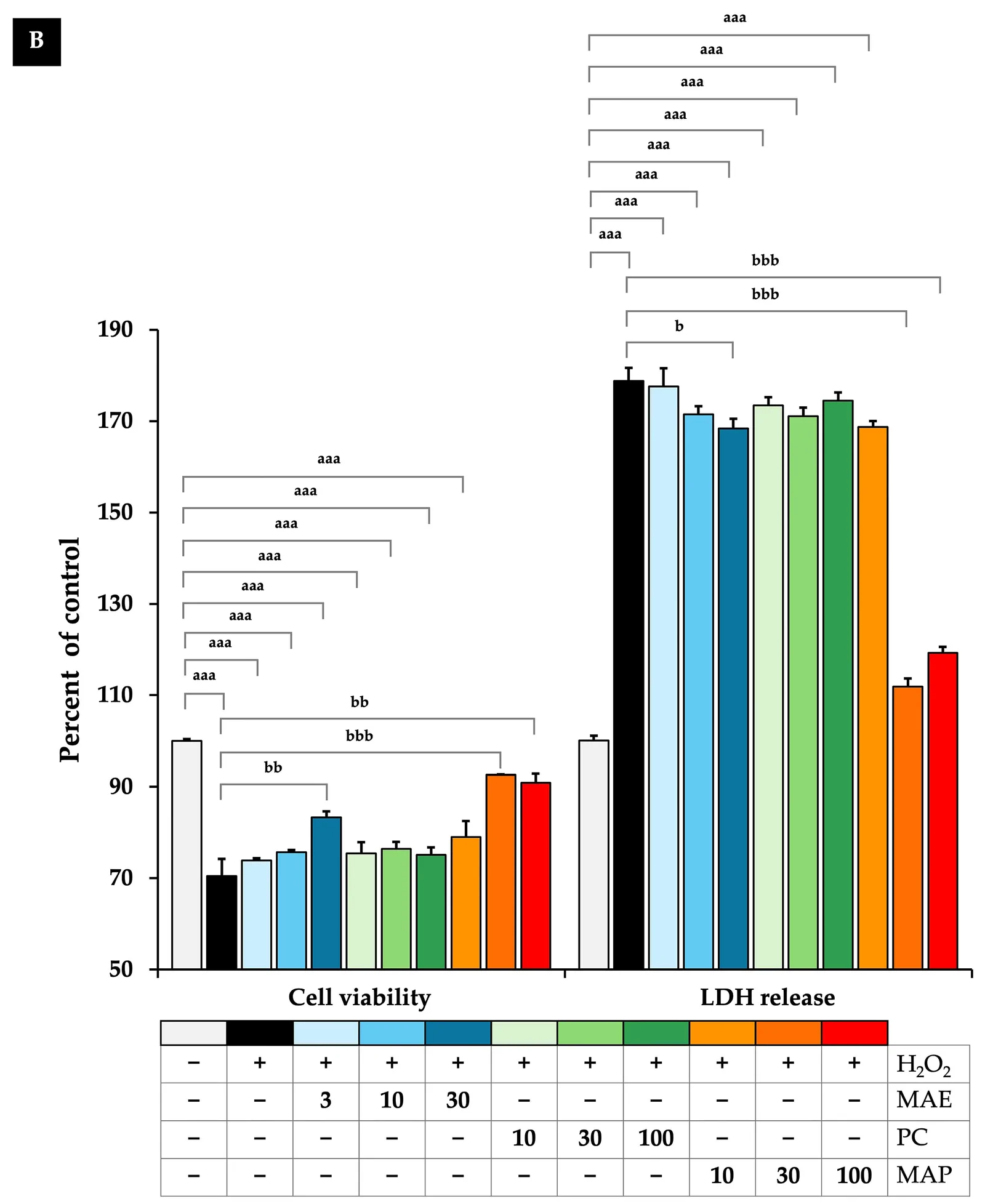
Effects of *Morus alba* L. fruit extract (MAE), phosphatidylcholine (PC), and *Morus alba* L. fruit extract phytosome (MAP) on cell viability and lactate dehydrogenase (LDH) release in H_2_O_2_-induced SH-SY5Y cell injury. (**A**) Representative images of SH-SY5Y cells observed under a phase-contrast microscope at 10× magnification. (**B**) Cell viability following treatment with MAE, PC, or MAP in the presence of H_2_O_2_. Data are presented as mean ± SEM from three independent experiments (*n* = 3). Statistical analysis was performed using one-way ANOVA followed by Tukey’s post hoc test. aaa indicates *p* < 0.001 compared with the untreated control group; b, bb, and bbb indicate *p* < 0.05, *p* < 0.01, and *p* < 0.001, respectively, compared with the H_2_O_2_-treated group.

Exposure of SH-SY5Y cells to H_2_O_2_ significantly reduced cell viability and increased LDH release compared with the control group (all *p* < 0.001) (Figure 3). Treatment with MAE at 30 μg/mL significantly increased cell viability and attenuated LDH release compared with the H_2_O_2_ + vehicle group (*p* < 0.01 and *p* < 0.05, respectively).

PC at all tested concentrations did not significantly improve cell viability or reduce LDH release compared with the H_2_O_2_ + vehicle group. In contrast, treatment with MAP at 30 and 100 μg/mL significantly restored cell viability (*p* < 0.001 and *p* < 0.01, respectively) and decreased LDH release (all *p* < 0.001) compared with H_2_O_2_ + vehicle group. Among the tested concentrations, MAP at 30 μg/mL produced the most pronounced increase in cell viability and reduction in LDH release.

Based on these findings, MAE and MAP at 30 μg/mL were selected for subsequent analyses and direct comparison of the extract and phytosome formulation at the same concentration.

### 3.5. Effects of MAP on HDAC Activity

**Figure 4:**
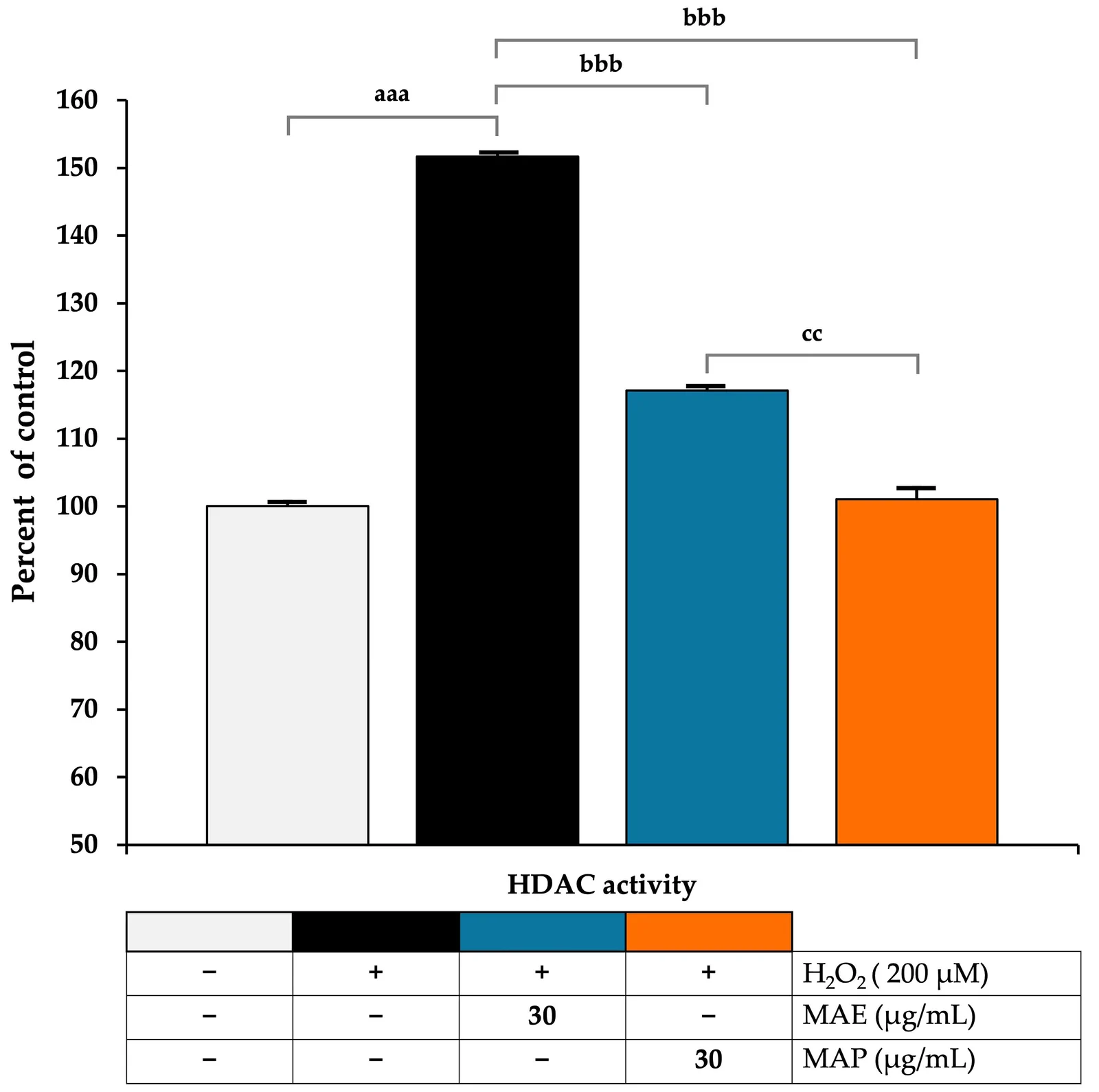
Effects of *Morus alba* L. fruit extract (MAE) and *Morus alba* L. fruit extract phytosome (MAP) on histone deacetylase (HDAC) activity in H_2_O_2_-induced SH-SY5Y cell injury. Data are presented as mean ± SEM from three independent experiments (*n* = 3). Statistical analysis was performed using one-way ANOVA followed by Tukey’s post hoc test. aaa indicates *p* < 0.001 compared with the untreated control group; bbb indicates *p* < 0.001 compared with the H_2_O_2_-treated group; cc indicates *p* < 0.01 compared with the MAE-treated group.

Following the observed protective effects against H_2_O_2_-induced cytotoxicity, the effects of MAE and MAP on HDAC activity were further evaluated (Figure 4). Exposure to H_2_O_2_ significantly increased HDAC activity compared with the control group (*p* < 0.001). Treatment with both MAE and MAP significantly reduced HDAC activity compared with the H_2_O_2_ + vehicle group (all *p* < 0.001). Notably, the MAP formulation produced a greater reduction in HDAC activity than MAE (*p* < 0.01).

### 3.6. Effects of MAP on SIRT1 Expression

**Figure 5:**
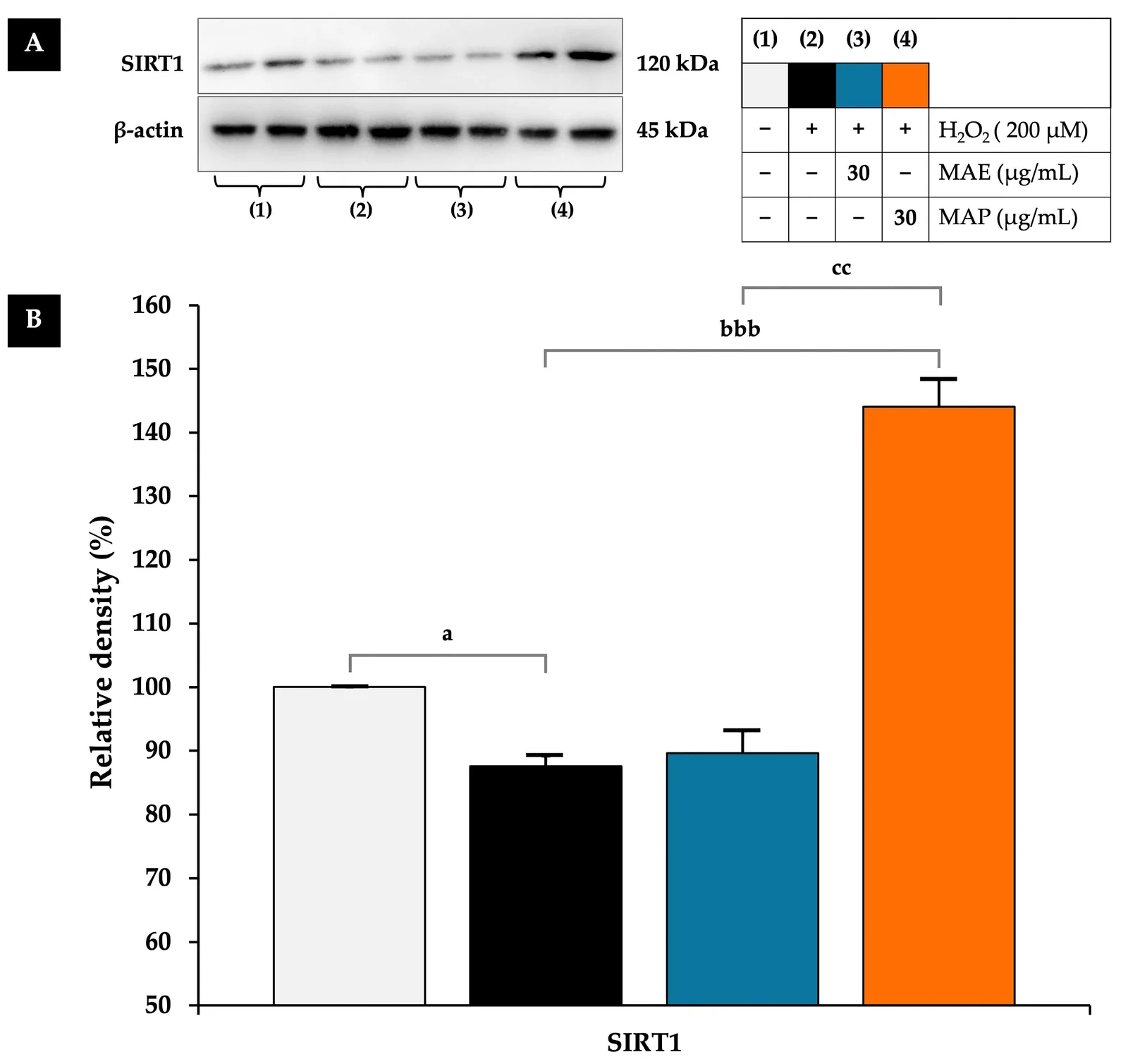
Effects of *Morus alba* L. fruit extract (MAE) and *Morus alba* L. fruit extract phytosome (MAP) on sirtuin 1 (SIRT1) expression in H_2_O_2_-induced SH-SY5Y cell injury. (**A**) Representative immunoblots of SIRT1 protein expression. (**B**) Quantitative analysis of SIRT1 protein expression normalized to β-actin. Data are presented as mean ± SEM from three independent experiments (*n* = 3). Statistical analysis was performed using one-way ANOVA followed by Tukey’s post hoc test. a indicates *p* < 0.05 compared with the untreated control group; bbb indicates *p* < 0.001 compared with the H_2_O_2_-treated group; cc indicates *p* < 0.001 compared with the MAE-treated group. (1) control; (2) H_2_O_2_ (200 μM) + vehicle; (3) H_2_O_2_ (200 μM) + MAE (30 μg/mL); and (4) H_2_O_2_ (200 μM) + MAP (30 μg/mL).

The effects of MAE and MAP on SIRT1 expression are shown in Figure 5. Exposure to H_2_O_2_ significantly decreased SIRT1 expression compared with the control group (*p* < 0.05). Treatment with MAP significantly increased SIRT1 expression compared with the H_2_O_2_ + vehicle group (*p* < 0.001). In addition, SIRT1 expression in the MAP-treated group was significantly higher than that in the MAE-treated group (*p* < 0.001). In contrast, treatment with MAE alone did not significantly alter SIRT1 expression compared with the H_2_O_2_ + vehicle group.

### 3.7. Effects of MAP on Oxidative Stress Markers

**Figure 6:**
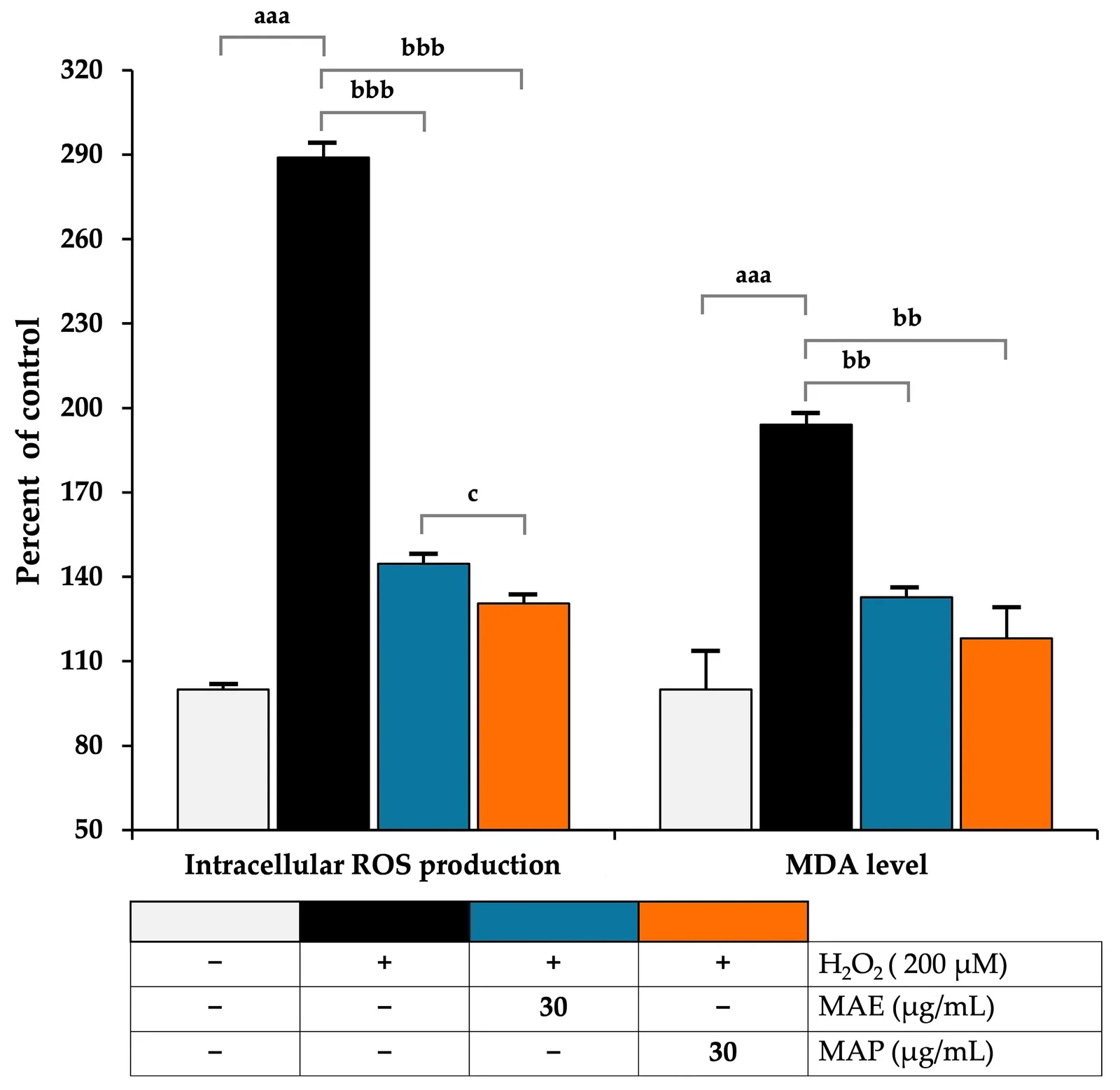
Effects of *Morus alba* L. fruit extract (MAE) and *Morus alba* L. fruit extract phytosome (MAP) on intracellular reactive oxygen species (ROS) production and malondialdehyde (MDA) levels in H_2_O_2_-induced SH-SY5Y cell injury. Data are presented as mean ± SEM from three independent experiments (*n* = 3). Statistical analysis was performed using one-way ANOVA followed by Tukey’s post hoc test. aaa indicates *p* < 0.001 compared with the untreated control group; bb and bbb indicate *p* < 0.01 and *p* < 0.001, respectively, compared with the H_2_O_2_-treated group; c indicates *p* < 0.05 compared with the MAE-treated group.

To further evaluate the effects of MAE and MAP on oxidative stress, intracellular ROS production and MDA levels were determined in H_2_O_2_-treated SH-SY5Y cells (Figure 6). Exposure to H_2_O_2_ significantly increased intracellular ROS production and MDA levels compared with the control group (*p* < 0.001 for both).

Treatment with both MAE and MAP significantly reduced intracellular ROS production compared with the H_2_O_2_ + vehicle group (all *p* < 0.001). Moreover, MAP produced a greater reduction in ROS levels than MAE (*p* < 0.05).

Similarly, treatment with MAE and MAP significantly decreased MDA levels compared with the H_2_O_2_ + vehicle group (both *p* < 0.01). However, no significant difference in MDA levels was observed between the MAE- and MAP-treated groups.

### 3.8. Effects of MAP on Antioxidant Enzyme Activities

**Figure 7:**
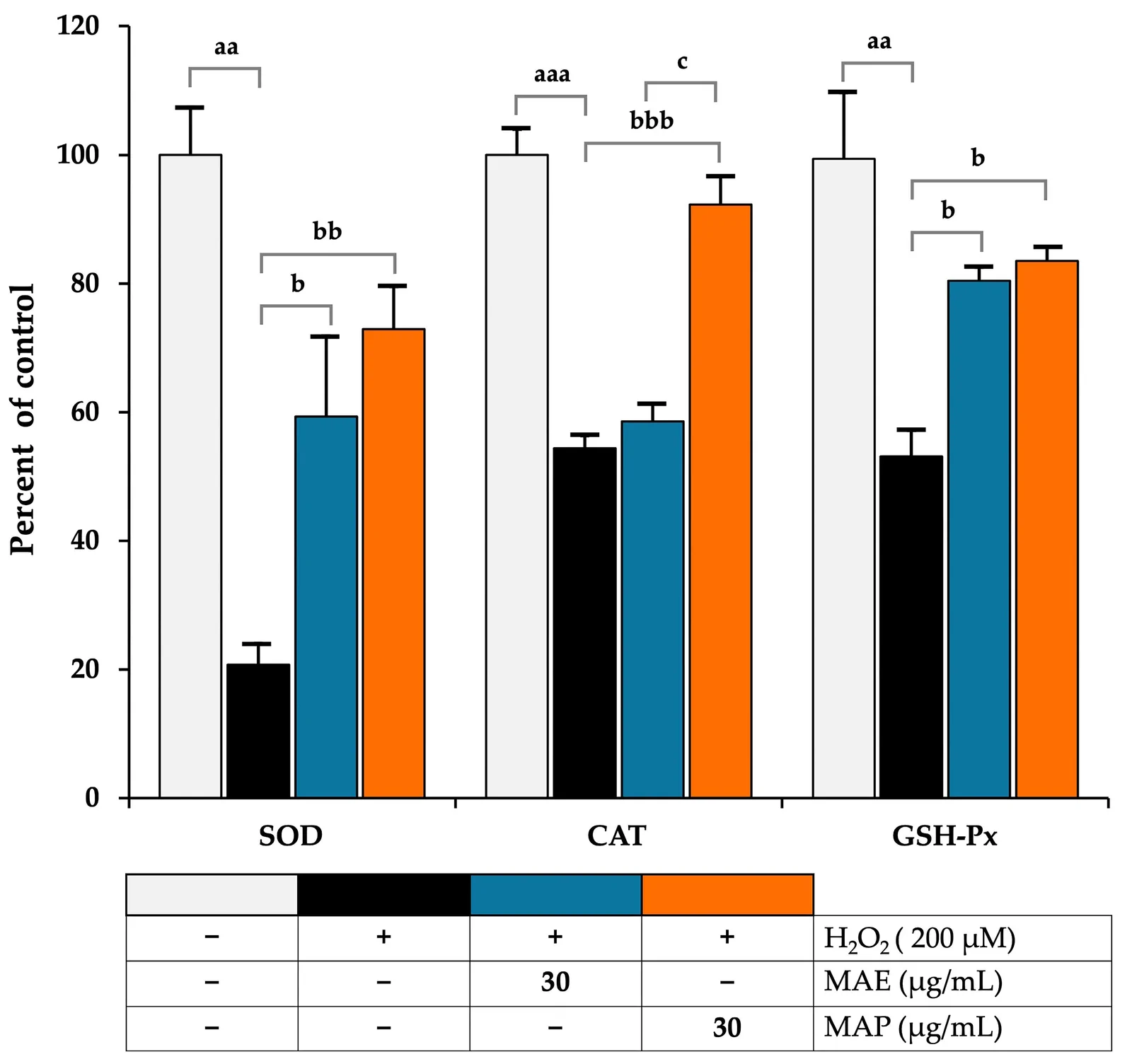
Effects of *Morus alba* L. fruit extract (MAE) and *Morus alba* L. fruit extract phytosome (MAP) on the activities of superoxide dismutase (SOD), catalase (CAT), and glutathione peroxidase (GSH-Px) in H_2_O_2_-induced SH-SY5Y cell injury. Data are presented as mean ± SEM from three independent experiments (*n* = 3). Statistical analysis was performed using one-way ANOVA followed by Tukey’s post hoc test. aa and aaa indicate *p* < 0.01 and *p* < 0.001, respectively, compared with the untreated control group; b, bb, and bbb indicate *p* < 0.05, *p* < 0.01, and *p* < 0.001, respectively, compared with the H_2_O_2_-treated group; c indicates *p* < 0.05 compared with the MAE-treated group.

Following the observed effects on oxidative stress markers, the activities of endogenous antioxidant enzymes were further evaluated in H_2_O_2_-treated SH-SY5Y cells (Figure 7). Exposure to H_2_O_2_ significantly reduced the activities of SOD, CAT, and GSH-Px compared with the control group (*p* < 0.01, *p* < 0.001, and *p* < 0.01, respectively).

Treatment with MAE significantly increased SOD and GSH-Px activities compared with the H_2_O_2_ + vehicle group (both *p* < 0.05), whereas no significant effect was observed on CAT activity. In contrast, treatment with MAP significantly increased the activities of SOD, CAT, and GSH-Px relative to the H_2_O_2_ + vehicle group (*p* < 0.01, *p* < 0.001, and *p* < 0.05, respectively). Furthermore, CAT activity was significantly higher in the MAP-treated group than in the MAE-treated group (*p* < 0.05).

### 3.9. Effects of MAP on Inflammatory Mediators

**Figure 8:**
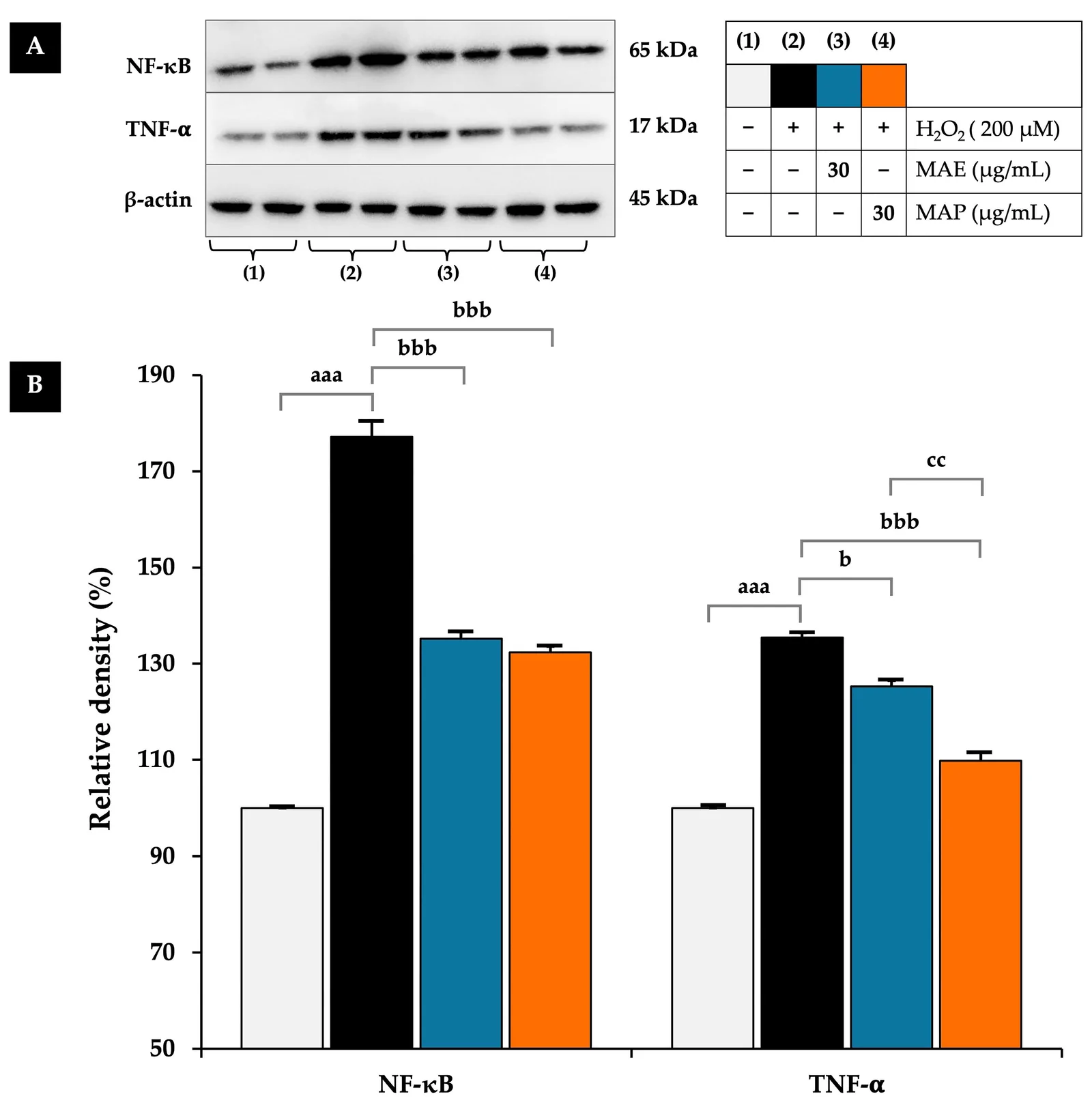
Effects of *Morus alba* L. fruit extract (MAE) and *Morus alba* L. fruit extract phytosome (MAP) on nuclear factor kappa B (NF-κB) and tumor necrosis factor-α (TNF-α) expression in H_2_O_2_-induced SH-SY5Y cell injury. (**A**) Representative immunoblots of NF-κB and TNF-α protein expression. (**B**) Quantitative analysis of NF-κB and TNF-α protein expression normalized to β-actin. Data are presented as mean ± SEM from three independent experiments (*n* = 3). Statistical analysis was performed using one-way ANOVA followed by Tukey’s post hoc test. aaa indicates *p* < 0.001 compared with the untreated control group; b and bbb indicate *p* < 0.05 and *p* < 0.001, respectively, compared with the H_2_O_2_-treated group; cc indicates *p* < 0.01 compared with the MAE-treated group. (1) control; (2) H_2_O_2_ (200 μM) + vehicle; (3) H_2_O_2_ (200 μM) + MAE (30 μg/mL); and (4) H_2_O_2_ (200 μM) + MAP (30 μg/mL).

Following the observed effects on oxidative stress markers and antioxidant enzyme activities, the expression of inflammatory mediators was further evaluated in H_2_O_2_-treated SH-SY5Y cells (Figure 8). Exposure to H_2_O_2_ significantly increased the expression of NF-κB and TNF-α compared with the control group (all *p* < 0.001).

Treatment with both MAE and MAP significantly reduced NF-κB expression compared with the H_2_O_2_ + vehicle group (all *p* < 0.001). Similarly, TNF-α expression was significantly decreased by treatment with MAE and MAP (*p* < 0.05 and *p* < 0.001, respectively). Furthermore, TNF-α expression was significantly lower in the MAP-treated group than in the MAE-treated group (*p* < 0.01).

### 3.10. Effects of MAP on Apoptosis-Related Protein Expression

**Figure 9:**
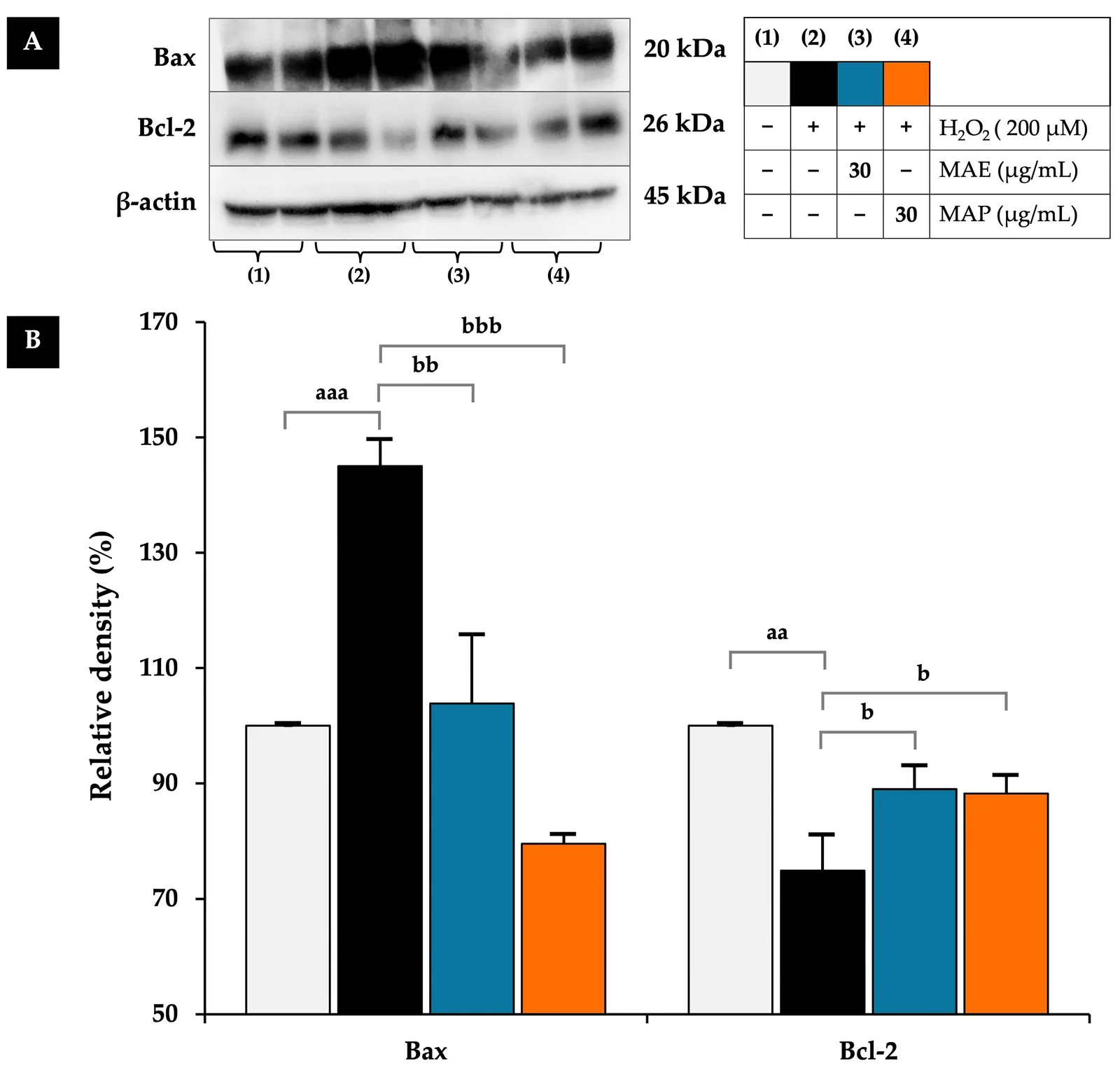
Effects of *Morus alba* L. fruit extract (MAE) and *Morus alba* L. fruit extract phytosome (MAP) on B-cell lymphoma 2 (Bcl-2) and Bcl-2-associated X protein (Bax) expression in H_2_O_2_-induced SH-SY5Y cell injury. (**A**) Representative immunoblots of Bcl-2 and Bax protein expression. (**B**) Quantitative analysis of Bcl-2 and Bax protein expression normalized to β-actin. Data are presented as mean ± SEM from three independent experiments (*n* = 3). Statistical analysis was performed using one-way ANOVA followed by Tukey’s post hoc test. aa and aaa indicate *p* < 0.01 and *p* < 0.001, respectively, compared with the untreated control group; b, bb, and bbb indicate *p* < 0.05, *p* < 0.01, and *p* < 0.001, respectively, compared with the H_2_O_2_-treated group. (1) control; (2) H_2_O_2_ (200 μM) + vehicle; (3) H_2_O_2_ (200 μM) + MAE (30 μg/mL); and (4) H_2_O_2_ (200 μM) + MAP (30 μg/mL).

Following the observed effects on oxidative stress and inflammatory mediators, the expression of apoptosis-related proteins was further evaluated in H_2_O_2_-treated SH-SY5Y cells (Figure 9). Exposure to H_2_O_2_ significantly increased Bax expression (*p* < 0.001) and decreased Bcl-2 expression (*p* < 0.01) compared with the control group.

Treatment with MAE significantly reduced Bax expression (*p* < 0.01) and increased Bcl-2 expression (*p* < 0.05) compared with the H_2_O_2_ + vehicle group. Similarly, treatment with MAP significantly decreased Bax expression (*p* < 0.001) and increased Bcl-2 expression (*p* < 0.05) compared with the H_2_O_2_ + vehicle group. No significant differences in Bax or Bcl-2 expression were observed between the MAE- and MAP-treated groups.

### 3.11. Molecular Docking Analysis of Anthocyanins with Target Proteins

Molecular docking analysis showed that both C3G and M3G were accommodated within the binding pockets of HDAC2, HDAC6, SIRT1, TNF-α, NF-κB, Bcl-2, and Bax, although their predicted binding scores and interaction patterns varied among the target proteins. The reliability of the docking protocol was evaluated by redocking the reference ligands into their corresponding protein binding sites. The resulting RMSD values for the seven protein–reference ligand complexes are presented in Table 3. In particular, the HDAC2 reference ligand showed an RMSD of 0.36 Å, indicating close agreement between the predicted and crystallographic binding poses. The predicted binding scores of C3G and M3G for the seven target proteins are summarized in Table 3, with more negative Vina scores indicating more favorable predicted binding under the applied docking conditions.

The two-dimensional interaction analysis further showed that both compounds formed multiple interactions with residues within their respective binding pockets (Table 4). C3G formed hydrogen-bonding interactions with several residues, including Arg197 and Arg311 in HDAC2; Asp122, Glu152, and Arg174 in HDAC6; Asp122, Ser442, Ser441, Ala262, and His363 in SIRT1; Tyr151, Leu120, and Ser60 in TNF-α; Glu89, Gln132, Asp125, Lys122, and Cys38 in NF-κB; Glu136 and Asp111 in Bcl-2; and Asp2 and Gln7 in Bax. Similarly, M3G formed hydrogen-bonding and hydrophobic interactions with residues across the seven target proteins, including Thr195 and Ser351 in HDAC2; Asp173, Arg174, Lys150, and Cys123 in HDAC6; Pro271, Asp318, and Gln201 in SIRT1; Thr151, Ser60, Leu120, and Gly122 in TNF-α; Glu89, Gln119, Gln132, Arg133, Cys38, and Lys37 in NF-κB; Ala149 and Phe153 in Bcl-2; and Met1 and Ser9 in Bax. Additional π-related interactions and hydrophobic contacts were also observed for both compounds within the respective binding sites.

The key protein–ligand interactions identified from the 2D docking interaction analysis are summarized in Table 5. Several residues were shared by C3G and M3G, whereas others were specific to each compound, reflecting differences in their interaction patterns across the target proteins.

## 4. Discussion

Anthocyanins from *Morus alba* L. fruit have been increasingly investigated as food-derived bioactive compounds with antioxidant, anti-inflammatory, and neuroprotective properties [28,29]. However, their application is limited by relatively poor physicochemical stability and bioavailability, which can restrict their biological effects [30]. Phospholipid-based delivery systems, including phytosomes, have therefore attracted interest as a means of improving the stability and functional availability of plant-derived bioactives [31,32,33]. In the present study, MAE was incorporated into a phosphatidylcholine-based phytosome (MAP). Among the formulations tested, the 50:50 MAE:PC ratio provided a favorable balance of anthocyanin association efficiency, particle size, polydispersity, and surface charge and was therefore selected for subsequent experiments. The MAP contained detectable levels of both C3G and M3G, with M3G showing a higher detected content than C3G under the applied HPLC-DAD conditions. These findings indicate that the MAP formulation retained the identified anthocyanin constituents while exhibiting physicochemical characteristics suitable for subsequent biological evaluation. Previous studies have reported that phytosome or phospholipid-based formulations can improve the stability and bioavailability of polyphenolic compounds, supporting the use of this approach for anthocyanin-rich extracts [31,32,33].

Anthocyanins from *Morus alba* L. fruit have previously shown neuroprotective activity in neuronal models [19,34,35]. C3G, in particular, has been reported to reduce oxidative injury and preserve cellular and mitochondrial function, while anthocyanins and their metabolites can attenuate ROS generation and apoptosis in SH-SY5Y cells exposed to H_2_O_2_ [19]. Consistent with these observations, H_2_O_2_ markedly impaired SH-SY5Y cell viability and increased LDH release in the present study, confirming substantial oxidative and membrane injury. MAE at 30 µg/mL partially protected the cells, whereas MAP produced a stronger protective response, particularly at 30 µg/mL. The greater activity of MAP despite comparison at the same nominal extract concentration suggests that phytosome formulation may have improved the functional availability of the extract rather than simply increasing the amount of anthocyanins administered. This interpretation is consistent with previous reports that phospholipid-based systems can enhance the biological performance of poorly bioavailable polyphenols, although intracellular uptake was not directly assessed in the present study [36,37].

Oxidative stress is closely associated with excessive ROS formation, lipid peroxidation, and disruption of endogenous antioxidant defenses [38]. Previous studies of mulberry anthocyanins have shown reductions in ROS and lipid peroxidation together with restoration of antioxidant enzymes in neuronal cells [9,19]. In agreement with these findings, both MAE and MAP reduced ROS and MDA levels following H_2_O_2_ exposure. MAP was more effective than MAE in reducing ROS, whereas their effects on MDA were comparable. This difference may indicate that phytosome formulation particularly enhanced the ability of anthocyanin-rich constituents to limit early intracellular oxidative burden, while suppression of downstream lipid peroxidation was already achieved effectively by MAE. The antioxidant response was further supported by changes in SOD, CAT, and GSH-Px. H_2_O_2_ suppressed all three enzymes, whereas MAE restored SOD and GSH-Px but not CAT. MAP restored all three antioxidant enzymes, with a significantly greater effect on CAT than MAE. Thus, the stronger restoration of enzymatic antioxidant defenses by MAP further suggests that the phytosome enhanced the biological activity of the extract under oxidative conditions.

The present findings also provide further insight into potential mechanisms underlying the effects of MAE beyond direct antioxidant activity. Oxidative stress can disturb acetylation homeostasis and alter the activity of HDACs, which have been implicated in neuronal survival and neurodegenerative processes [39,40]. At the same time, SIRT1 has been reported to protect neuronal cells against oxidative stress and to suppress downstream inflammatory and apoptotic signaling [41,42,43]. In the present study, H_2_O_2_ increased HDAC activity and reduced SIRT1 expression, whereas both MAE and MAP reduced HDAC activity and MAP markedly restored SIRT1 expression. The greater effect of MAP on both parameters was associated with greater modulation of these stress-responsive acetylation-related responses. However, because the HDAC assay measured overall HDAC activity rather than individual HDAC isoforms, and because direct regulatory interactions were not experimentally demonstrated, these findings should be interpreted as evidence of modulation of acetylation-related responses rather than proof of a specific HDAC–SIRT1 pathway. Moreover, the present study cannot determine whether these changes represent primary effects of MAP or secondary responses following the attenuation of oxidative stress.

Oxidative stress is also closely linked to neuroinflammatory signaling, particularly through NF-κB and TNF-α [44,45,46]. Anthocyanins have been reported to suppress NF-κB activation and downstream inflammatory mediators in neuronal and other cellular models, while studies of *Morus alba* L. have similarly described anti-inflammatory effects associated with NF-κB-related signaling [9,47]. Consistent with this literature, H_2_O_2_ increased NF-κB and TNF-α expression, whereas both MAE and MAP reduced these inflammatory mediators. MAP produced a greater reduction in TNF-α than MAE, indicating that the phytosome formulation may enhance the anti-inflammatory activity of the extract. This response is particularly relevant because persistent oxidative and inflammatory signaling can reinforce neuronal injury, suggesting that the protective activity of MAP may involve coordinated regulation of redox and inflammatory responses rather than ROS scavenging alone.

Apoptotic signaling was similarly affected. H_2_O_2_ increased the pro-apoptotic protein Bax and decreased the anti-apoptotic protein Bcl-2, consistent with activation of mitochondrial apoptotic signaling under oxidative stress [19,21,22,24]. Previous studies have shown that anthocyanins can attenuate oxidative-stress-associated apoptosis in neuronal models [19,48]. In agreement with these reports, both MAE and MAP decreased Bax and increased Bcl-2 expression. Although MAP showed a numerically stronger effect on Bax, the differences between MAE and MAP were not statistically significant for Bax or Bcl-2. Therefore, the enhanced cytoprotective activity provided by MAP cannot be attributed solely to stronger modulation of these apoptotic proteins and may instead be related to the combined effects on oxidative stress, antioxidant defenses, inflammatory signaling, and deacetylation-related responses.

The molecular docking results provide preliminary structural insights consistent with these biological observations. C3G and M3G showed favorable predicted interactions with HDAC2, HDAC6, SIRT1, TNF-α, NF-κB, Bcl-2, and Bax, involving hydrogen bonding together with hydrophobic and π-mediated interactions. The low RMSD obtained during redocking of the reference ligands supports the reliability of the docking protocol. The predicted interactions with proteins involved in oxidative stress, inflammation, and apoptosis suggest potential molecular interactions that may be relevant to the biological activities observed experimentally. However, docking represents a computational prediction of ligand–protein recognition and does not establish direct binding or functional modulation. Moreover, the extract contains multiple constituents, and the biological effects of MAE and MAP cannot be attributed exclusively to C3G or M3G on the basis of the docking results. Further studies using direct binding assays and pathway-specific interventions would therefore be required to establish whether these predicted interactions occur experimentally and whether they contribute functionally to the observed biological effects.

Several limitations should be considered when interpreting these findings. First, the study was performed in an in vitro SH-SY5Y cell model, which does not fully reproduce the complexity of neuronal responses or the pharmacokinetic and metabolic conditions encountered in vivo. Accordingly, the present findings should be interpreted as evidence of potential neuroprotective activity rather than a demonstrated neuroprotective effect in vivo. Second, although HPLC-DAD confirmed the presence of C3G and M3G in MAE and MAP, the analysis did not provide comprehensive phytochemical profiling, and matrix-specific recovery of these anthocyanins from the MAP formulation was not directly assessed. Thus, the contribution of individual anthocyanins and other unidentified constituents to the observed biological effects could not be distinguished. Third, intracellular uptake, anthocyanin stability, cellular availability, and in vivo absorption and metabolism were not evaluated. Fourth, the observed changes in HDAC activity and SIRT1 expression indicate modulation of deacetylation-related responses but do not establish a direct regulatory relationship between these targets. Similarly, the molecular docking results provide preliminary structural insights into potential interactions of C3G and M3G with proteins involved in oxidative stress, inflammation, and apoptosis, but these predicted interactions require experimental validation. Future studies using comprehensive phytochemical and matrix-specific recovery analyses, cellular uptake and bioavailability assessments, target-specific interventions, appropriate animal models, and ultimately human intervention studies are needed to clarify the mechanisms, in vivo biological relevance, and potential application of MAP as a functional food ingredient.

## 5. Conclusions

Overall, the MAE exhibited protective effects against H_2_O_2_-induced oxidative injury in SH-SY5Y cells, and the MAP formulation enhanced several aspects of this activity. The enhanced response of MAP was most evident in ROS suppression, restoration of antioxidant defenses, attenuation of TNF-α, and modulation of HDAC activity and SIRT1 expression, whereas its effects on lipid peroxidation and apoptotic proteins were comparable to those of MAE. These findings suggest that the MAP formulation may enhance the protective activity of MAE in this cellular model and provide in vitro evidence supporting the potential of MAP as a food-derived source of bioactive anthocyanins. Further studies using animal models and, ultimately, clinical investigations are warranted to validate the neuroprotective potential of MAP, establish its biological relevance in vivo, and clarify whether HDAC/SIRT1-related responses contribute causally to the observed biological effects.

## Figures and Tables

**Table 1:** Physicochemical characterization of MAP.

MAE:PC	TPC (µg GAE/mg)	TAC (µg C3G Equivalent/mg)	AE (%)	PS (nm)	ZP (mV)	PDI
100:0	135.67 ± 3.50	72.33 ± 2.83	–	–	–	–
25:75	33.67 ± 0.84	16.83 ± 0.35	85.07 ± 5.51	114.02 ± 1.58	−30.24 ± 0.33	0.12 ± 0.0011
33:67	42.67 ± 0.96	22.33 ± 0.54	85.15 ± 3.91	135.10 ± 2.52	−32.63 ± 0.53	0.17 ± 0.0009
50:50	67.67 ± 0.84	35.33 ± 0.48	82.96 ± 4.53	153.80 ± 3.51	−38.54 ± 0.32	0.18 ± 0.0022
67:33	88.00 ± 1.53	44.50 ± 0.67	69.81 ± 3.23	173.84 ± 6.07	−23.80 ± 1.02	0.20 ± 0.0016
75:25	98.23 ± 1.07	49.33 ± 0.67	54.98 ± 2.72	204.70 ± 7.35	−18.81 ± 0.45	0.26 ± 0.0192

Data are presented as mean ± SEM from three independent experiments (*n* = 3). MAE, *Morus alba* L. fruit extract; PC, phosphatidylcholine; TPC, total phenolic content; TAC, total anthocyanin content; AE, anthocyanin association efficiency; PS, particle size; ZP, zeta potential; PDI, polydispersity index.

**Table 2:** Validation parameters of the developed HPLC-DAD method.

Parameters	C3G	M3G
LOD (µg/mL)	0.05	0.10
LOD (S/N)	3.01 ± 0.03	3.31 ± 0.43
LOQ (µg/mL)	0.25	0.50
LOQ (S/N)	10.10 ± 0.61	10.27 ± 0.15
Linear range (µg/mL)	0.05–10	0.10–5
Regression equation	y = 7.899x − 6.848	y = 5.442x − 1.895
R^2^	0.999	0.995
Repeatability (%RSD)	0.08–2.1	1.42–2.93
Intermediate precision (%RSD)	0.13–2.30	0.16–2.28
Recovery (%) Low	105.42 ± 0.79	109.53 ± 1.63
Recovery (%) Medium	97.70 ± 1.16	91.61 ± 0.72
Recovery (%) High	108.03 ± 0.66	101.11 ± 1.35
MAE (µg /mg extract)	13.15 ± 0.04	38.57 ± 0.05
MAP (µg /mg formulation)	6.72 ± 0.01	17.84 ± 0.02

Data are presented as mean ± SD from three independent experiments (*n* = 3). LOD, limit of detection; LOQ, limit of quantification; S/N, signal-to-noise ratio; R^2^, coefficient of determination; %RSD, relative standard deviation.

**Table 3:** Predicted binding scores (Vina score; kcal/mol) and RMSD of the investigated compounds and reference ligands for the selected target proteins obtained using AutoDock Vina.

Compounds	HDAC2 (4LXZ)	HDAC6 (5EDU)	SIRT1 (4ZZJ)	TNF-α (2AZ5)	NF-κB (1VKX)	Bcl-2 (6O0K)	Bax (1F16)
**Investigated compounds**							
C3G	−8.6	−10.0	−10.6	−9.1	−7.0	−7.9	−9.9
M3G	−9.1	−8.7	−10.2	−9.5	−7.6	−7.6	−8.7
**Reference ligands (RMSD)**							
Vorinostat	0.36	–	–	–	–	–	–
Trichostatin A	–	0.82	–	–	–	–	–
EX-527	–	–	1.02	–	–	–	–
Thalidomide	–	–	–	0.87	–	–	–
Sulindac	–	–	–	–	0.74	–	–
Venetoclax	–	–	–	–	–	0.17	–
Eltrombopag	–	–	–	–	–	–	0.96

C3G, cyanidin-3-O-glucoside; M3G, malvidin-3-O-glucoside; HDAC2, histone deacetylase 2; HDAC6, histone deacetylase 6; SIRT1, sirtuin 1; TNF-α, tumor necrosis factor-alpha; NF-κB, nuclear factor kappa B; Bcl-2, B-cell lymphoma 2; Bax, Bcl-2-associated X protein; RMSD, root-mean-square deviation.

**Table 4:** Two-dimensional interaction profiles of C3G and M3G with the selected target proteins.

Target Proteins	C3G	M3G
HDAC2 (4LXZ)	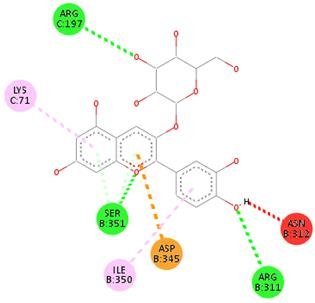	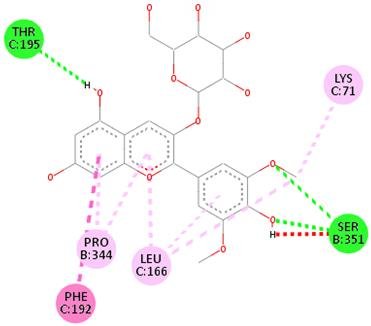
HDAC6 (5EDU)	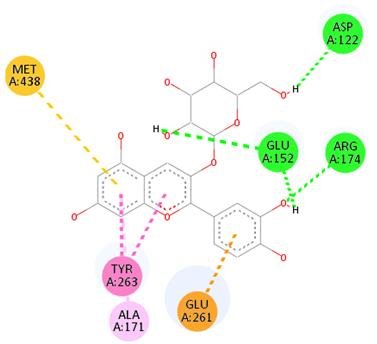	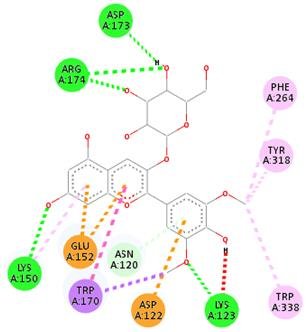
SIRT1 (4ZZJ)	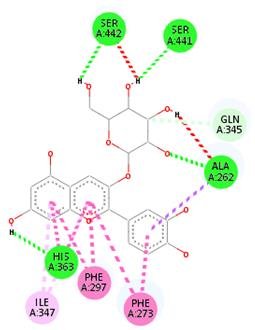	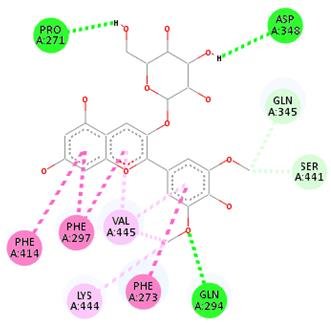
TNF-α (2AZ5)	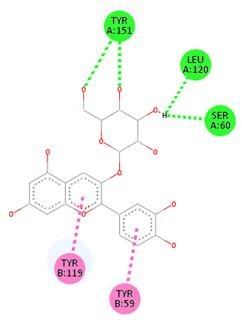	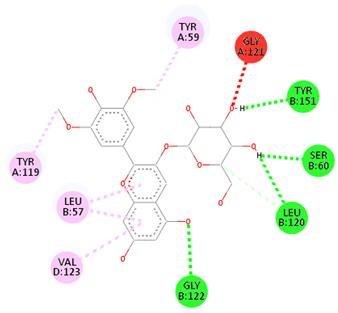
NF-κB (1VKX)	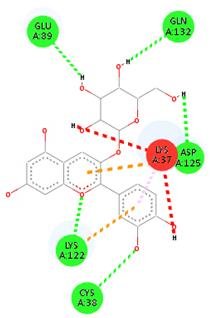	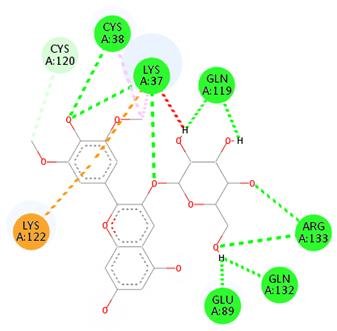
Bcl-2 (6O0K)	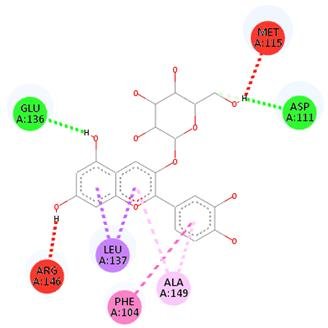	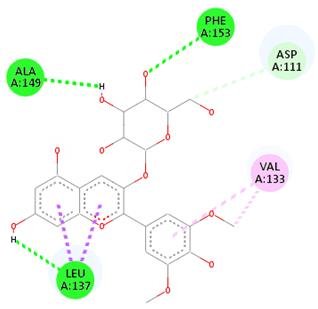
Bax (1F16)	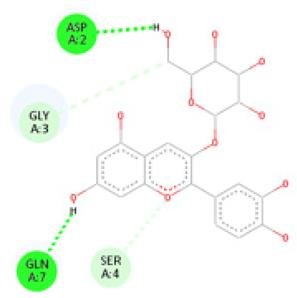	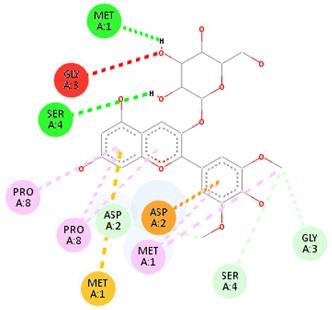

C3G, cyanidin-3-O-glucoside; M3G, malvidin-3-O-glucoside; HDAC2, histone deacetylase 2; HDAC6, histone deacetylase 6; SIRT1, sirtuin 1; TNF-α, tumor necrosis factor-alpha; NF-κB, nuclear factor kappa B; Bcl-2, B-cell lymphoma 2; Bax, Bcl-2-associated X protein.

**Table 5:** Key protein–ligand interactions identified from the two-dimensional docking interaction analysis.

Target Protein	Compounds	Hydrogen Bonds	π-Related Interactions	Hydrophobic Interactions	Unfavorable Interactions
HDAC2	C3G	Arg197; Arg311	Ser351 (π-donor H-bond); Asp345 (π-anion)	Lys71; Ile350 (π-alkyl)	Asn312
HDAC6	C3G	Asp122; Glu152; Arg174	Glu261 (π-anion); Met438 (π-sulfur); Tyr263 (π–π T-shaped)	Ala171 (alkyl)	—
SIRT1	C3G	Asp122; Ser442; Ser441; Ala262; His363	Gln345 (π-donor H-bond)	Ile347 (π-alkyl); Phe297; Phe273 (π–π T-shaped)	Gln345/Ala262
TNF-α	C3G	Tyr151; Leu120; Ser60	Tyr119 (π–π T-shaped); Tyr59 (π–π T-shaped)	—	—
NF-κB	C3G	Glu89; Gln132; Asp125; Lys122; Cys38	Lys37; Lys122 (π-cation)	—	Lys37
Bcl-2	C3G	Glu136; Asp111	—	Leu137; Ala149 (π-alkyl); Phe104 (π–π T-shaped)	Met115; Arg146
Bax	C3G	Asp2; Gln7	Gly3; Ser4 (π-donor H-bond)	—	—
HDAC2	M3G	Thr195; Ser351	Pro344; Phe192 (π–π T-shaped)	Lys71; Leu166 (π-alkyl)	Ser351
HDAC6	M3G	Asp173; Arg174; Lys150; Cys123	Asn120 (π-donor H-bond); Glu152; Asp122 (π-anion)	Trp170; Phe265; Tyr318; Trp338 (π-alkyl)	—
SIRT1	M3G	Pro271; Asp318; Gln201	Gln395; Ser411 (π-donor H-bond)	Phe419; Phe297; Phe273; Tyr318; Trp338; Ile415; Lys144 (π/alkyl)	—
TNF-α	M3G	Thr151; Ser60; Leu120; Gly122	—	Tyr39; Tyr119; Leu57; Val123 (π-alkyl)	Gly121
NF-κB	M3G	Glu89; Gln119; Gln132; Arg133; Cys38; Lys37	Cys120 (π-donor H-bond); Lys122 (π-cation)	—	Lys37
Bcl-2	M3G	Ala149; Phe153	Asp111 (π-donor H-bond)	Leu137; Val133 (π-alkyl)	—
Bax	M3G	Met1; Ser9	Asp2 (π-anion); Ser4 (π-donor H-bond)	Met1; Pro8; Pro3 (π-alkyl)	Gly3

C3G, cyanidin-3-O-glucoside; M3G, malvidin-3-O-glucoside; HDAC2, histone deacetylase 2; HDAC6, histone deacetylase 6; SIRT1, sirtuin 1; TNF-α, tumor necrosis factor-alpha; NF-κB, nuclear factor kappa B; Bcl-2, B-cell lymphoma 2; Bax, Bcl-2-associated X protein.

## Data Availability

The original contributions presented in this study are included in the article/Supplementary Materials. Further inquiries can be directed to the corresponding author.

## Supplementary Materials

The following supporting information can be downloaded at https://www.mdpi.com/article/10.3390/foods15203664/s1, Figure S1: Original, unprocessed, and uncropped full-length membrane images from Western blot analysis of SIRT1 expression with β-actin as the internal loading control; Figure S2: Original, unprocessed, and uncropped full-length membrane images from Western blot analysis of NF-κB and TNF-α expression with β-actin as the internal loading control; Figure S3: Original, unprocessed, and uncropped full-length membrane images from Western blot analysis of Bax and Bcl-2 expression with β-actin as the internal loading control; Figure S4: Representative phase-contrast microscopic images of SH-SY5Y cells from the four experimental groups at 10× magnification.
