# Supplementary material for: Anthocyanin-Rich Morus alba L. Fruit Extract Phytosome Protects Neuronal Cells Against Oxidative Stress-Induced Cytotoxicity
Source: Foods. 2026 Oct 15;15(20):3664. doi: 10.3390/foods15203664 (PMC13704223; doi:10.3390/foods15203664)
Supplement: Supplementary file 1 [file foods-15-03664-s001.zip › foods-4601863-supplementary.pdf]

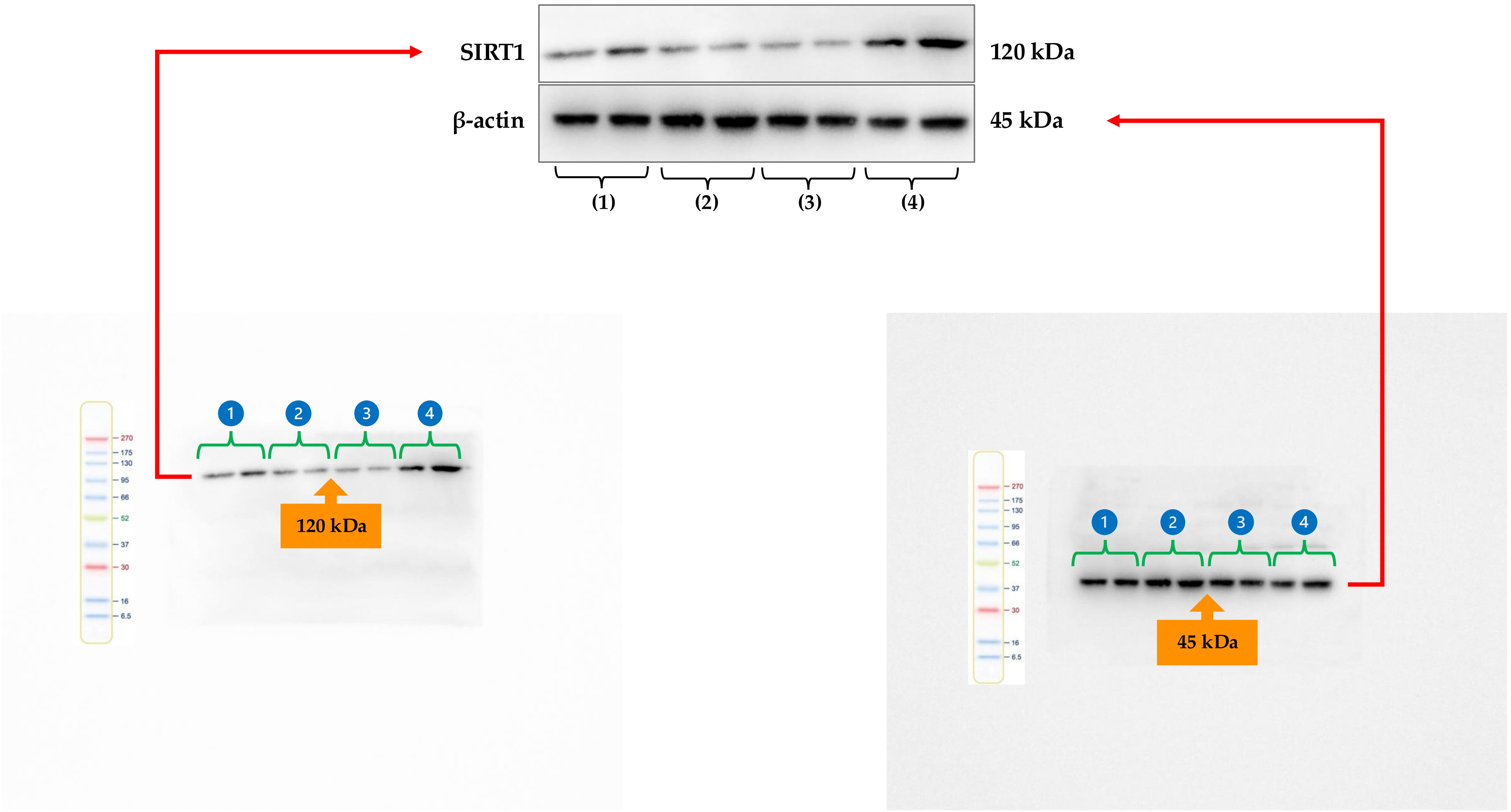

Figure S1: Original, unprocessed, and uncropped full-length membrane images from Western blot analysis of SIRT1 expression, with β-actin as the internal loading control. The experimental groups were as follows: (1) control, (2) H_2O_2 + vehicle, (3) H_2O_2 + MAE, and (4) H_2O_2 + MAP. H_2O_2, hydrogen peroxide; SIRT1, sirtuin 1; β-actin, beta-actin; MAE, *Morus alba* L. fruit extract at 30 μg/mL; MAP, *Morus alba* L. fruit extract phytosome at 30 μg/mL.

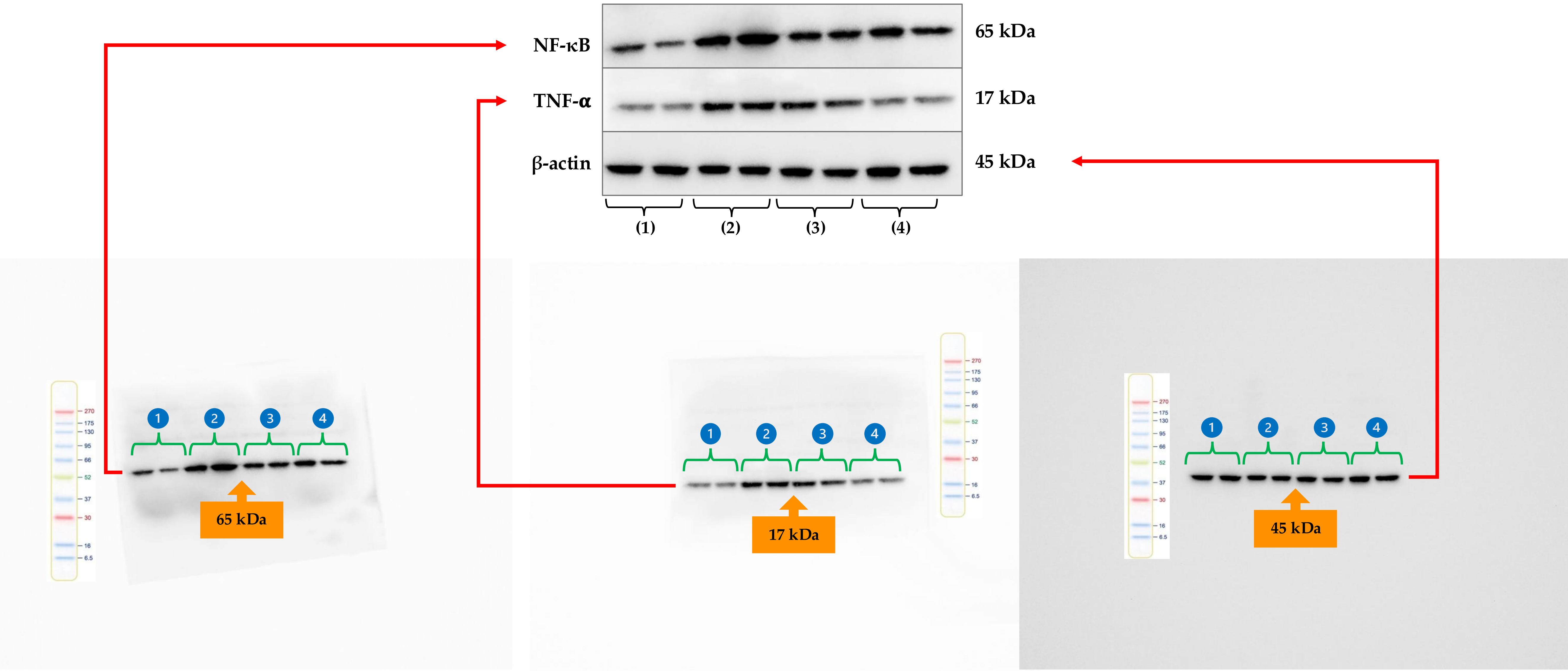

Figure S2: Original, unprocessed, and uncropped full-length membrane images from Western blot analysis of NF-κB and TNF-α expression, with β-actin as the internal loading control. The experimental groups were as follows: (1) control, (2) H_2O_2 + vehicle, (3) H_2O_2 + MAE, and (4) H_2O_2 + MAP. H_2O_2, hydrogen peroxide; NF-κB, nuclear factor kappa B; TNF-*α*, tumor necrosis factor-alpha; β-actin, beta-actin; MAE, *Morus alba* L. fruit extract at 30 µg/mL; MAP, *Morus alba* L. fruit extract phytosome at 30 µg/mL.

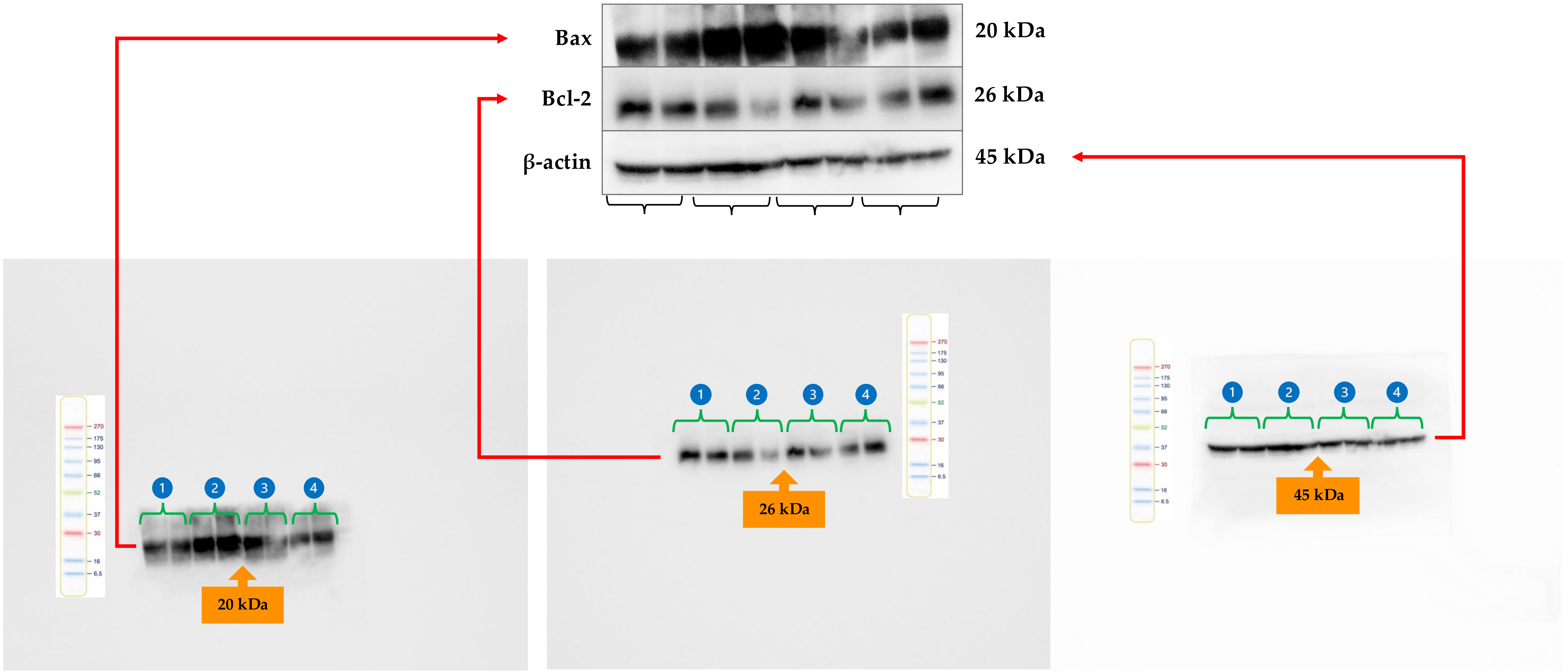

Figure S3: Original, unprocessed, and uncropped full-length membrane images from Western blot analysis of Bax and Bcl-2 expression, with β-actin as the internal loading control. The experimental groups were as follows: (1) control, (2) H_2O_2 + vehicle, (3) H_2O_2 + MAE, and (4) H_2O_2 + MAP. H_2O_2, hydrogen peroxide; Bax, Bcl-2-associated X protein; Bcl-2, B-cell lymphoma 2; β-actin, beta-actin; MAE, *Morus alba* L. fruit extract at 30 μg/mL; MAP, *Morus alba* L. fruit extract phytosome at 30 μg/mL.

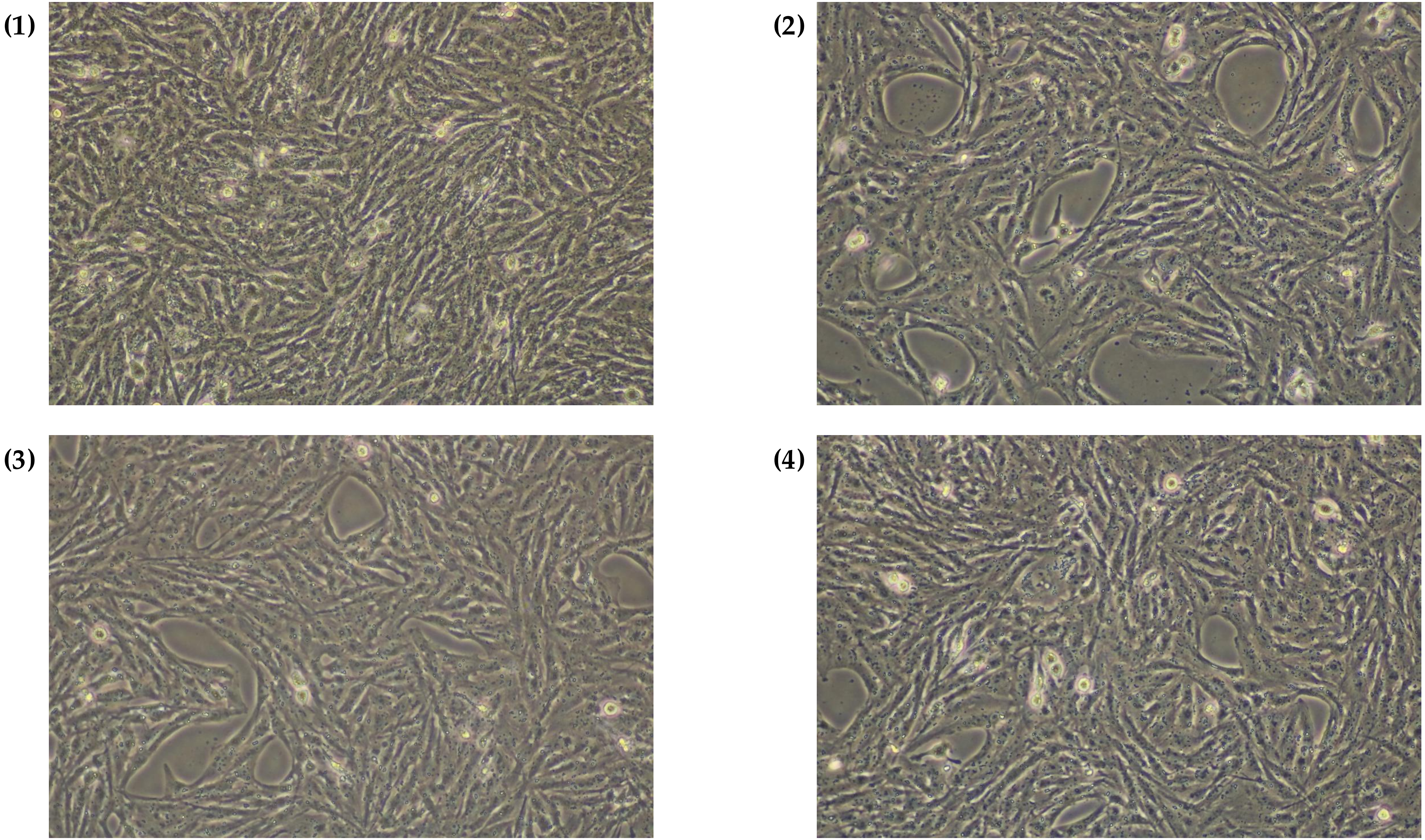

Figure S4: Representative phase-contrast microscopic images of SH-SY5Y cells from the four experimental groups at 10× magnification. The experimental groups were as follows: (1) control, (2) H_2O_2 + vehicle, (3) H_2O_2 + MAE, and (4) H_2O_2 + MAP. H_2O_2, hydrogen peroxide; MAE, *Morus alba* L. fruit extract at 30 μg/mL; MAP, *Morus alba* L. fruit extract phytosome at 30 μg/mL.
